# Resistance Determinants and Their Genetic Context in Enterobacteria from a Longitudinal Study of Pigs Reared under Various Husbandry Conditions

**DOI:** 10.1128/AEM.02612-20

**Published:** 2021-03-26

**Authors:** Dominic Poulin-Laprade, Jean-Simon Brouard, Nathalie Gagnon, Annie Turcotte, Alexandra Langlois, J. Jacques Matte, Catherine D. Carrillo, Rahat Zaheer, Tim A. McAllister, Edward Topp, Guylaine Talbot

**Affiliations:** a Sherbrooke Research and Development Centre, Agriculture and Agri-Food, Sherbrooke, QC, Canada; b Ottawa Research and Development Centre, Canadian Food Inspection Agency, Ottawa, ON, Canada; c Lethbridge Research and Development Centre, Agriculture and Agri-Food, Lethbridge, AB, Canada; d London Research and Development Centre, Agriculture and Agri-Food, London, ON, Canada; University of Helsinki

**Keywords:** ESBL, *Enterobacteriaceae*, *Escherichia coli*, antibiotic susceptibility testing, coselection, conjugation, multidrug resistance, pig, swine, whole-genome sequencing

## Abstract

Pigs are major reservoirs of resistant *Enterobacteriaceae* that can reach humans through consumption of contaminated meat or vegetables grown in manure-fertilized soil. Samples were collected from sows during lactation and from their piglets at five time points spanning the production cycle. Cefotaxime-resistant bacteria were quantified and isolated from feed, feces, manures, and carcasses of pigs reared with penicillin-using or antibiotic-free husbandries. The isolates were characterized by antibiotic susceptibility testing, whole-genome sequencing, and conjugation assays. The extended-spectrum β-lactamase (ESBL) phenotype was more frequent in isolates originating from antibiotic-free animals, while the bacteria isolated from penicillin-using animals were on average resistant to a greater number of antibiotics. The ESBL-encoding genes identified were *bla*_CTX-M-1_, *bla*_CTX-M-15_, and *bla*_CMY-2_, and they colocalized on plasmids with various genes encoding resistance to β-lactams, co-trimoxazole, phenicols, and tetracycline, all antibiotics used in pig production. Groups of genes conferring the observed resistance and the mobile elements disseminating multidrug resistance were determined. The observed resistance to β-lactams was mainly due to the complementary actions of penicillin-binding proteins, an efflux pump, and β-lactamases. Most resistance determinants were shared by animals raised with or without antimicrobials. This suggests a key contribution of indigenous enterobacteria maternally transmitted along the sow lineage regardless of antimicrobial use. It is unclear whether the antimicrobial resistance observed in the enterobacterial populations of the commercial pig herds studied was present before the use of antibiotics or the extent to which historical antimicrobial use exerted a selective pressure defining the resistant bacterial populations in farms using penicillin prophylaxis.

**IMPORTANCE** Antimicrobial resistance is a global threat that needs to be fought on numerous fronts along the One Health continuum. Vast quantities of antimicrobials are used in agriculture to ensure animal welfare and productivity and are arguably a driving force for the persistence of environmental and foodborne resistant bacteria. This study evaluated the impact of conventional, organic, and other antibiotic-free husbandry practices on the frequency and nature of antimicrobial resistance genes and multidrug-resistant enterobacteria. It provides knowledge about the relative contribution of specific resistance determinants to observed antibiotic resistance. It also shows the clear coselection of genes coding for extended-spectrum beta-lactamases and genes coding for resistance to antibiotics commonly used for prophylaxis or in curative treatments in pig operations.

## Abstract (precis)

Antimicrobial resistance is a global threat that needs to be fought on numerous fronts along the One Health continuum. Vast quantities of antimicrobials are used in agriculture to ensure animal welfare and productivity and are arguably a driving force for the persistence of environmental and foodborne resistant bacteria.

## INTRODUCTION

Antimicrobial resistance (AMR) jeopardizes the treatment of infections afflicting humans, pets, livestock, and plants. Blame is often directed toward livestock producers, urging them to modify husbandry practices to reduce antimicrobial usage, improve the quality of life for the animals, and improve their environmental footprint. Pork producers with organic (1) and certified-humane (2) certifications are therefore gaining ground, but these are still marginal production practices in Europe and the Americas (3). Producers and veterinarians are concerned with animal welfare in antibiotic-free operations, as antibiotics are critical for the treatment of bacterial infections that could also potentially threaten food safety (4). Previous studies showed a reduced prevalence of antibiotic-resistant bacteria in animals with restricted antibiotic use (5, 6). A study involving pigs reared in nine European countries showed that the abundance of macrolide and tetracycline resistance genes in bacteria isolated from feces coincided with the amounts of macrolides and tetracyclines administered, but this association was not observed for β-lactams (7).

In 2018, antibiotics saved the lives of at least 17,000 Canadians, and the effects of AMR on labor productivity reduced Canada’s gross domestic product by an estimated $2 billion representing about 0.13% of the country’s economy (8). The bulk of antibiotics are administered to livestock, presumably promoting the development and spread of antibiotic resistance genes in foodborne pathogens. Among the latter are the extended-spectrum β-lactamase (ESBL)-producing *Enterobacteriaceae*, which are considered a serious threat to human health (9, 10). ESBL contribute to resistance against β-lactams, including penicillins and third-generation cephalosporins (3GC). In Canada, 3GC are considered of very high importance in human medicine (11) and their use is decreasing due to federal legislation (12). Cefotaxime and ceftiofur are both 3GC and are commonly used in humans and pigs to treat recalcitrant bacterial infections.

Many public health agencies worldwide are transitioning from culture-dependent antibiotic susceptibility testing (AST) to whole-genome sequencing (WGS) for surveillance. WGS can provide a complete overview of the antibiotic resistance gene (ARG) arsenal of a pathogen and the nature of propagating mobile elements in the clinic and identify their transmission routes through food and the environment. The accuracy of gene-based AMR prediction is generally high (13–17), but there is still a need for acquiring knowledge concerning the relative contribution of the resistance determinants to the observed antibiotic resistance.

The overarching goal of this study was to verify whether rearing pigs with antibiotic-free practices reduces the abundance of resistant bacteria in pig feces and carcasses. To do so, we sampled antibiotic-free farms that complied with three types of certifications: (i) organic (antibiotic-free 1, AF1), (ii) certified-humane (antibiotic-free 2, AF2), and (iii) AGRO-COM (antibiotic-free 3, AF3). The latter is a verification of claims allowing the commercial partners to display optional antibiotic-free claims on their products (18). It ensures compliance with protocol and meets the requirements of the Canadian Food Inspection Agency (19, 20). The AF1, AF2, and AF3 husbandries all abide by the verification of the “antibiotic-free” claim. AF2 farms were also certified humane (2) and provided rearing on straw and reduced animal density compared to conventional practices. AF1 farms were not only certified humane but also organic (1) and involved feeding the pigs with organic grain. As a control group in the same geographical location, we sampled a conventional farm (CV) that uses prophylaxis penicillin for farrowing sows and piglets.

Specifically, the following three objectives were undertaken: (i) quantification of the occurrence of cefotaxime (CTX)-, meropenem (MER)-, and tetracycline (TET)-resistant *Enterobacteriaceae* in pigs at various production stages (sows, suckling, weaning, growing, and finishing pigs and their carcasses) reared in commercial facilities that vary in their use of antibiotics from conventional to none at all (Table 1), (ii) genotypic and phenotypic characterization of 3GC-resistant *Enterobacteriaceae* and Acinetobacter species isolated from feeds, feces, manure, and carcass surfaces, and (iii) identification of most probable genetic determinants conferring and propagating the observed antibiotic resistance. Overall, the comprehensive characterization of porcine CTX-resistant isolates allowed identification of various plasmid clusters with cargo ESBL-encoding genes colocalizing with other genes conferring resistance to antibiotics commonly used in pig production and humans.

**TABLE 1:** Distinctive practices of the four husbandries studied

Husbandry^*a*^	Antibiotic^*b*^	Feed	Certified-humane^*d*^	Outdoor access	Weaning age (days)
AF1	None	Organic	Yes	Yes^*c*^	28
AF2	None		Yes	No	28
AF3	None		No	No	21
CV	Penicillin	Medicated	No	No	21

a The husbandries included penicillin-using (conventional [CV]) and three antibiotic-free practices certified as organic (antibiotic-free 1 [AF1] [1]), certified-humane (antibiotic-free 2 [AF2] [2]), and AGRO-COM (antibiotic-free 3 [AF3] [18]).

b Antibiotic used in prophylaxis.

c The animals had outdoor access for one day during the fattening period.

d See reference 112.

## RESULTS

### Phenotypic resistance in *Enterobacteriaceae* from feed, feces, and manure.

**FIG 1:**
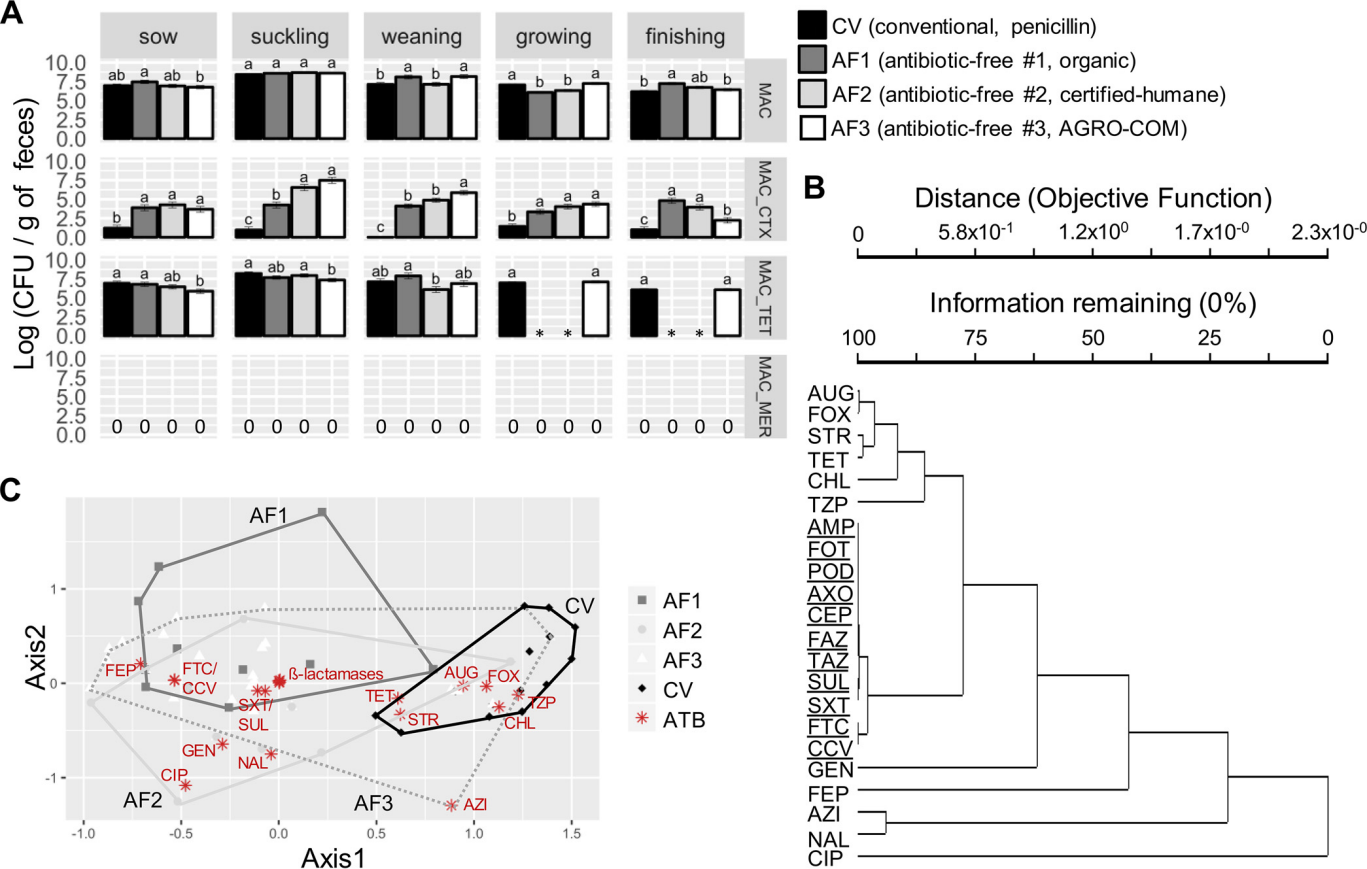
Antibiotic-resistant *Enterobacteriaceae* according to animal type and husbandry. (A) The bars represent the least-square means plus or minus standard error of mean for log transformations of CFU per gram of feces on MacConkey agar plates (MAC) without supplementation or supplemented with cefotaxime (_CTX), tetracycline (_TET), or meropenem (_MER). One-way analysis of variance (ANOVA) with Tukey’s multiple comparisons test was used to compare the least-square means from the same animal type reared in the different husbandries. Different letters on top of bars indicate significantly different results (*P < *0.01). Missing data are indicated by asterisks, while zeroes mark the absence of colonies. (B) Hierarchical cluster analysis based on Bray-Curtis distances of the AST profiles with group average as the linkage method between the antibiotics tested on 247 bacterial strains isolated from feces. Antibiotics of the β-lactam/sulfamethoxazole-trimethoprim cluster are underlined. (C) Nonmetric multidimensional scaling (NMS) plot of the AST profiles of 247 isolates from feces of animals reared in all four husbandries. Stars represent antibiotics as indicated, while geometric shapes represent the grouping of individual isolates. The antibiotics tested are as follows: ampicillin (AMP), amoxicillin-clavulanic acid (Augmentin; AUG), piperacillin-tazobactam (TZP), cefazolin (FAZ), cephalothin (CEP), cefotaxime (CTX), cefpodoxime (POD), ceftazidime (TAZ), ceftriaxone (CRO), cefotaxime-clavulanic acid (FTC), ceftazidime-clavulanic acid (CCV), cefepime (FEP), cefoxitin (FOX), imipenem (IMP), meropenem (MER), sulfisoxazole (SUL), trimethoprim-sulfamethoxazole (SXT), tetracycline (TET), gentamicin (GEN), streptomycin (STR), azithromycin (AZI), ciprofloxacin (CIP), nalidixic acid (NAL), and chloramphenicol (CHL).

The abundances of total *Enterobacteriaceae* and those resistant to CTX, TET, and MER were assessed by plating feed, feces, and manure samples on selective media. The fecal samples from suckling piglets originating from all farm types had the most abundant *Enterobacteriaceae*. TET-resistant (TET^R^) colonies were frequently isolated from primary samples regardless of husbandry practice (Fig. 1A and Table 1). Surprisingly, the frequency of CTX-resistant *Enterobacteriaceae* in feces was higher in the three antibiotic-free settings than in the CV setting (Fig. 1A). According to a repeated-measures analysis, the abundance of total *Enterobacteriaceae* decreased with age, from suckling to finishing stages, in all animal groups (*P* < 0.0001), and the absolute abundance of CTX- and TET-resistant *Enterobacteriaceae* also decreased with age for certified-humane (AF2) and AGRO-COM (AF3) husbandries (*P* < 0.0001). Viable-cell counts were also carried out on feed samples collected in trolleys near the pens of the sampled animals over the course of the project. Low counts of presumptive CTX-resistant *Enterobacteriaceae* were obtained solely for one CV group in feed given to growing and finishing pigs (Fig. S1). Presumptive CTX-resistant *Enterobacteriaceae* were found in the majority of manure samples collected from tank and pen floors and were collectively referred to as manure (Fig. S2). Meropenem resistance was not observed in any feed or fecal samples but was encountered occasionally in manure samples recovered from a CV farm housing weaning pigs.

### Resistance profiles of isolates.

A collection of ∼10,000 isolates was generated and screened for resistance to the 3GC CTX and ceftriaxone (CRO). Resistant bacteria were more abundant in AF1 and AF2, while CV isolates were more susceptible (Fig. S3).

To evaluate the antibiotic resistance profiles of enterobacteria resistant or susceptible to CTX across the production continuum, a collection of 359 isolates from feed, feces, manure, and carcass swabs were tested for susceptibility to 24 antibiotics belonging to 14 drug classes (Fig. S4 and Table S1). The CTX-susceptible (CTX^S^) isolates were on average resistant to three antibiotics, while the CTX-resistant (CTX^R^) isolates were on average resistant to 10 antibiotics. The resistance to ampicillin (AMP) in CTX^S^ isolates from CV farms was four times greater than that in antibiotic-free farms. As expected, nearly all the isolates initially selected on MacConkey agar plates supplemented with CTX (MAC-CTX) were resistant to AMP, first-generation cephalosporins (1GC), and 3GC, regardless of the husbandry. Resistance to sulfonamides, in combination with a diaminopyrimidine or not, was also encountered in most CTX^R^ isolates. Surprisingly, while only 4% of CTX^R^ isolates from CV farms exhibited the ESBL phenotype, 70% of the CTX^R^ isolates originating from antibiotic-free farms displayed the ESBL phenotype, as the MIC for combinations of CTX or ceftazidime (TAZ) with clavulanic acid was at least 3 logs lower than the MIC for CTX or TAZ alone. In contrast, 99% of the CV CTX^R^ isolates were also resistant to cefoxitin (FOX) and TET, compared to only 23% and 40%, respectively, in antibiotic-free settings. Furthermore, CV CTX^R^ isolates were, respectively, 2-, 12-, and 15-fold more frequently resistant to streptomycin (STR), piperacillin-tazobactam (TZP), and chloramphenicol (CHL) than CTX^R^ isolates from antibiotic-free farms. Resistance to TET and STR was also more prevalent in CV isolates following primary isolation on antibiotic-free MAC agar. Resistance to gentamicin (GEN), cefepime (FEP), azithromycin (AZI), and fluoroquinolones was scarce. Only Providencia rettgeri isolates from one manure sample from the certified-humane husbandry were resistant to impenem (IMP) and MER, but this species may have elevated MIC to carbapenems by mechanisms other than production of carbapenemases (21). Indeed, these isolates were not actively excreting carbapenemases as verified by the modified carbapenem inactivation method (mCIM) (Table S2).

To reveal close associations between resistances to major antibiotic classes, a clustering analysis of the AST profiles of fecal isolates was performed (Fig. 1B). This analysis clearly illustrates the close linkage between resistance to β-lactams and sulfonamide-diaminopyrimidine in the three antibiotic-free settings. In addition, nonmetric multidimensional scaling of the AST profiles of fecal isolates illustrates that those belonging to the three antibiotic-free settings tend to be more alike and distinct from the CV profile (Fig. 1C), even though a multiresponse permutation procedure showed that the AST profiles associated with each husbandry were different from each other (*P* values < 0.001) for all pairwise comparisons between the four husbandries. In contrast with the isolates sampled from feces and manure that were predominantly Escherichia coli, isolates from carcasses consisted mostly of Enterobacter cloacae and Acinetobacter species (Table S1). Accordingly, the AST profiles observed for carcass isolates were distinct from those observed from feces and manure isolates (Fig. S4 and Table S1).

### Resistome of isolates.

**FIG 2:**
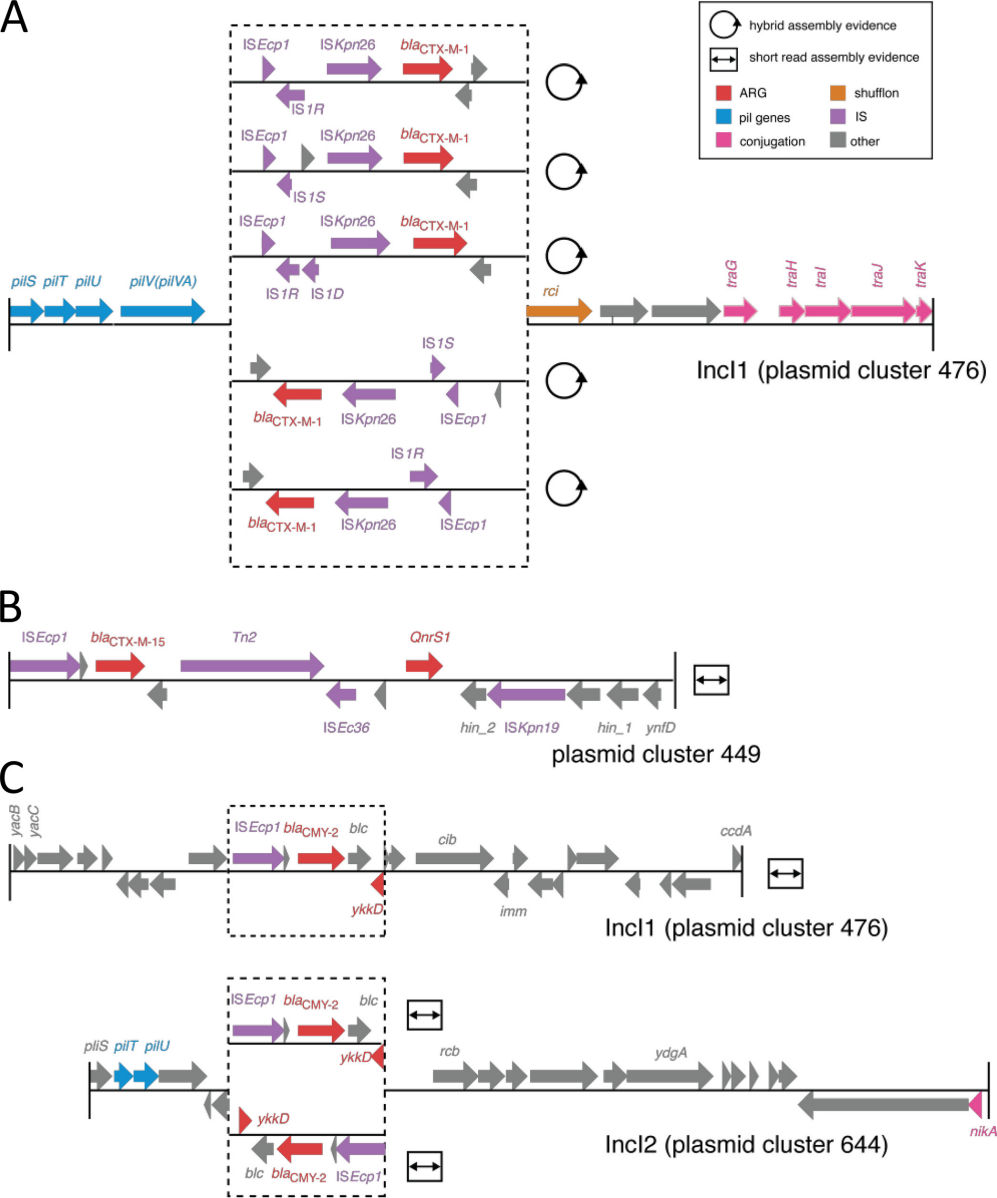
Genetic context of ESBL-encoding genes. Gene organization surrounding *bla*_CTX-M-1_ (A), *bla*_CTX-M-15_ (B), and *bla*_CMY-2_ (C) as computed by GENcontext.

Whole-genome sequencing using short- and long-read technologies allowed detection of ARGs and the plasmid content of the isolates. The ESBL-encoding genes encountered were *bla*_CTX-M-1_, *bla*_CMY-2_, and *bla*_CTX-M-15_. *bla*_CTX-M-1_ was the most frequent, with 107 occurrences, was invariably associated with IncI1 plasmids of the plasmid cluster 476, IS*Ec*p1, IS*Kpn*26, and members of the IS1 family, and was observed only in antibiotic-free husbandries (Fig. 2A, Fig. S5, and Table S1). There were nine occurrences of *bla*_CTX-M-15_ originating from organic animals and located on the chromosome or on ColRNAI plasmids and surrounded by IS*Ecp*1, IS*Ec*36, IS*Kpn*19, and *Tn2* (Fig. 2B). The gene *bla*_CMY-2_ was found in 50 instances in all four husbandries, was associated with IS*Ec*p1 and *ykkD*, which encodes the subunit of an efflux pump, and was found on chromosomes or on IncI1, IncI2, IncF, IncA/C2, or ColRNAI plasmids (Fig. 2C). The ESBL-encoding genes did not occur in isolates originating from carcass swabs. The genes coding for other β-lactamases also differed depending on whether the isolates were from feces-manure or carcass samples (Table S1).

**FIG 3:**
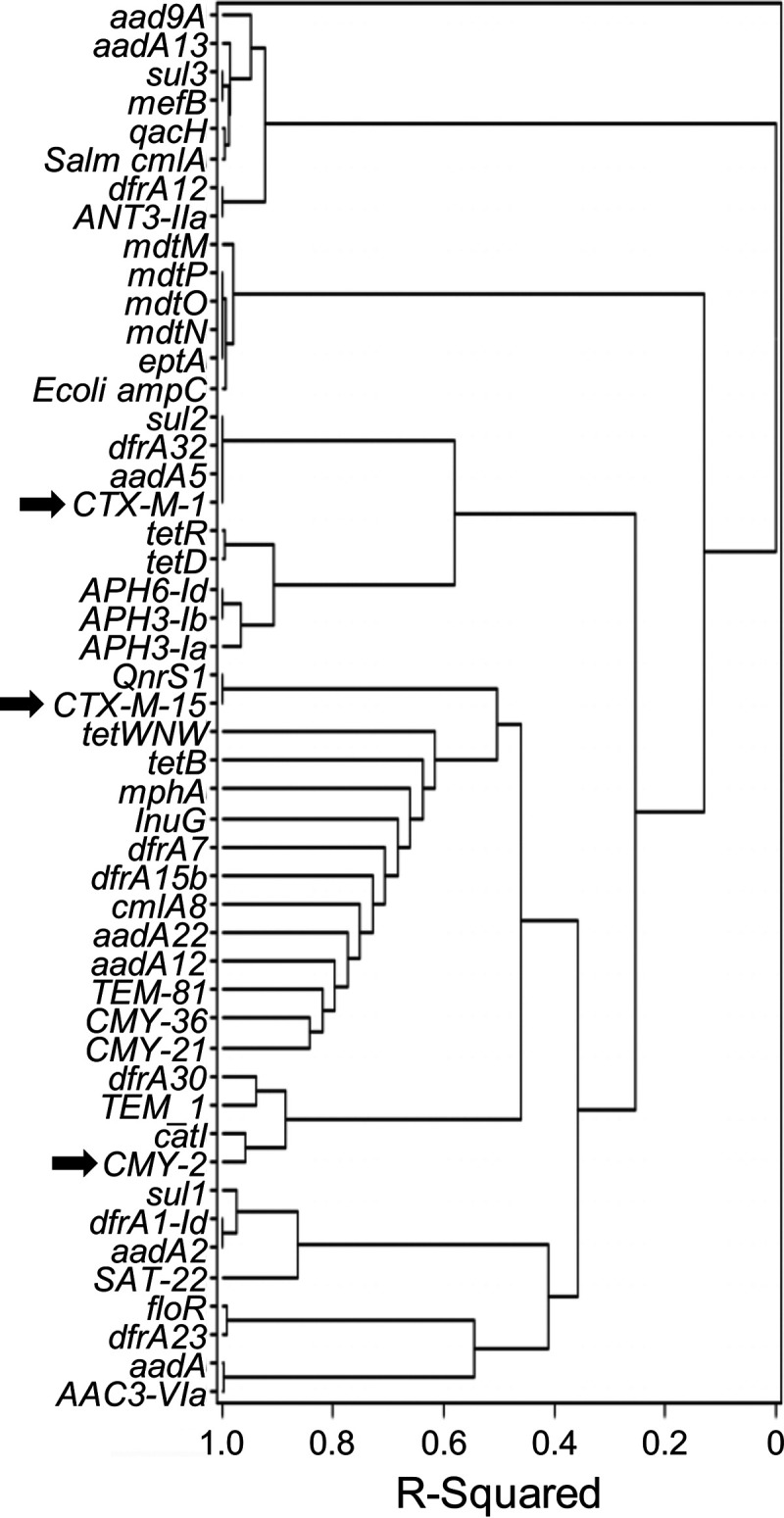
Colocalization of ARGs on plasmids. Hierarchical clustering based on Bray-Curtis distances using the Ward method for tree construction on 269 items, where an item is a unique combination of a feces isolate with one of its plasmids, and the 49 ARG encountered. ESBL-encoding genes are indicated by arrows.

ARGs putatively conferring resistance to aminoglycosides, carbapenems, diaminopyrimidines, sulfonamides, fluoroquinolones, macrolides, and tetracyclines were also detected (Table S1). The observed likelihood of colocalization of ARGs on the same plasmid is illustrated by a clustering analysis based on Bray-Curtis distances (Fig. 3). Generally, a locus of genes conferring resistance to co-trimoxazole (*sul2* and *drfA*) and streptomycin (*aadA5*) and associated with IS*26* and other IS elements was located approximately 80 kbp from *bla*_CTX-M-1_ on the same plasmid. The genes *bla*_CTX-M-15_ and *bla*_CMY-2_ were, respectively, associated with *qnrS1*, a gene conferring resistance to fluoroquinolones, and *catI*, a gene conferring resistance to phenicols, but also to an array of other genes conferring resistance to cephalosporins and other β-lactams, aminoglycosides, trimethoprim, and tetracycline. The *bla*_IMP-7_ gene, putatively coding for a metallo-β-lactamase, was identified in Providencia rettgeri isolated from manure. This gene was surrounded by the *sul2*, *aad5*, and *APH(3′')-Ia* ARGs and by the *xerC*, *mobA*, and *repA* genes involved in recombination, plasmid conjugation, and plasmid replication, respectively.

### Concordance between resistance genotype and phenotype.

**FIG 4:**
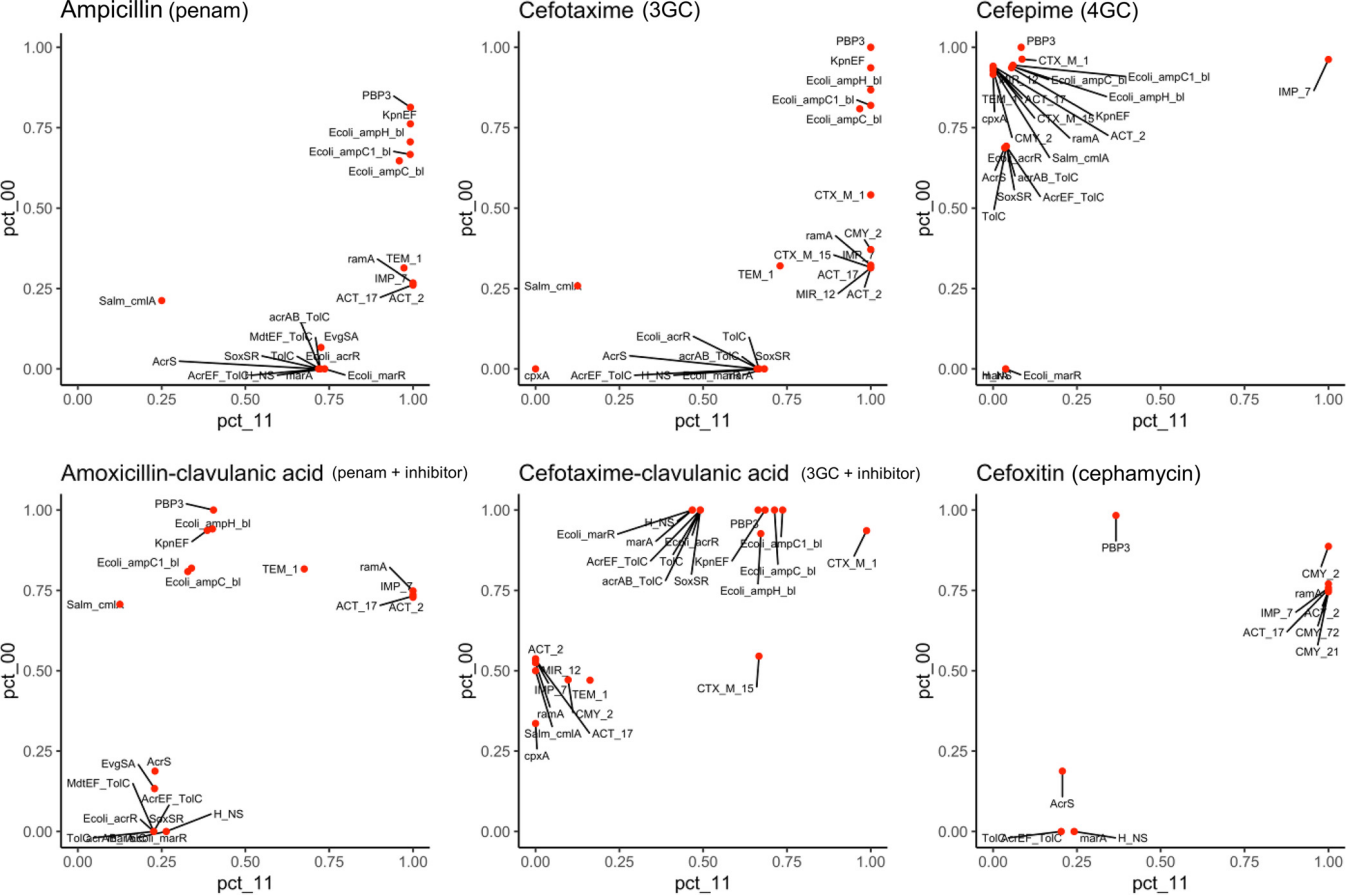
Concordance between the resistome and the resistance observed by antibiotic susceptibility testing of isolates. The dots represent individual genes, and their position is at the intersection between the percentage of susceptible isolates without the gene (pct_00) and the percentage of resistant isolates with the gene (pct_11). The antibiotics tested and the associated antibiotic classes are indicated on top of the graphs. Detailed descriptions of the genes are located in Table S1. Results for the 18 other antibiotics tested are shown in Fig. S6.

The concordance between genotypes and phenotypes for porcine isolates was calculated for 24 antibiotics (Table S1) and was used to infer the relative importance of genetic determinants for the resistance phenotypes observed. Penicillins were routinely used in the CV sows and piglets included in this study, and the observed resistance to ampicillin and first- and third-generation cephalosporins was mainly due to the complementary actions of the penicillin-binding proteins PBP3 and AmpH, the KpnEF efflux pump, and chromosomally encoded AmpC β-lactamases (Fig. 4). These resistance determinants were aided by the β-lactamases TEM-1, IMP-7, and ACT encoded by genes present on plasmids in ∼30% of the strains. Interestingly, *ramA*, which codes for a transcriptional factor regulating the expression of the efflux pump AcrAB-TolC and the porin OmpF, coincides with the resistance to ampicillin as much as the mobile β-lactamases. When a β-lactamase inhibitor was used in combination with amoxicillin, a penam similar to ampicillin, the resistance determinants PBP3, *kpnEF*, and *amp* remained important, but *ramA*, *bla*_IMP-7_, and *bla*_ACT_ displayed an increased contribution to resistance as the percentage of “gene not present/strain susceptible” profiles rose from 25% to 75% (Fig. 4). A second combination of a penam with a β-lactamase inhibitor, piperacillin-tazobactam, resulted in *bla*_ACT-2_ being the main resistance determinant (Fig. S6). When 3GC were tested in conjunction with a β-lactamase inhibitor, *bla*_CTX-M-1_ was the resistance determinant of utmost importance, but the AcrAB and AcrEF efflux pumps, PBP3, KpnEF, and Amp also contributed to the observed resistance. As expected, *bla*_CMY-2_ was the leading determinant conferring resistance to cefoxitin, a cephamycin (Fig. 4). The *bla*_IMP-7_ gene was associated with resistance to the fourth-generation cephalosporin (4GC) cefepime and carbapenems (Fig. 4 and Fig. S6). The resistance determinant for azithromycin was *mphA*, while for nalidixic acid it was a mutated *gyrA* (22), and the *cmlA*, *catI*, and *floR* genes were identified in chloramphenicol-resistant isolates. The tetracycline resistance determinants included *tetA*, *tetB*, and *tetWNW* and were most likely under the control of the TetD and TetR transcriptional regulators. As for the resistance to sulfonamide-diaminopyrimidine, *sul2* and *dfrA32* genes accompanied by *kpnEF* were identified.

### Plasmidome of isolates.

The 1,526 plasmids hosted in the sequenced bacterial isolates were categorized into clusters of similar plasmids based on their predicted replicase, relaxase, and mating pair formation types (23). A total of 187 distinct plasmid clusters were identified with diversity in frequencies, size, relaxase, mpf types, predicted mobility, and ARG content (Tables S3 and S4). A correspondence analysis using the 27 plasmid clusters found in feces illustrates that most ARG-encoding plasmids were common to all husbandries and stages of the pork production cycle (Fig. S7). The predominant ARG-encoding plasmids belonged to an IncI1 plasmid cluster named 476 by MOB-suite, whose representatives were detected for all husbandries and at every point of the swine production cycle, with the exception of carcasses. Nearly all members of cluster 476 harbored ESBL-encoding genes (79% possess *bla*_CTX-M-1_ in antibiotic-free settings only, 20% encode *bla*_CMY-2_ mostly in CV but also in antibiotic-free settings) and other ARGs, including *bla*_TEM-1_, *aadA5*, *sul2*, and *dfrA17* (Fig. S5).

A total of 54 distinct plasmid clusters colocalized in isolates containing a 476 plasmid, with up to 12 plasmid clusters observed in a single isolate. In addition to plasmid cluster 476, 19 other plasmid clusters carried genes coding for β-lactamases (Fig. S5). Interestingly, IncI2 representatives of cluster 644 were associated with CV husbandry, and 11 of the 16 plasmids identified harbored *bla*_CMY-2_. The plasmid cluster 449 invariably contained *bla*_CTX-M-15_ and was detected in only one group of organic animals. Plasmids found in feces-manure samples were markedly different from those isolated from carcasses (Table S1).

### Transfer of ESBL-encoding conjugative plasmids.

CTX resistance was transferable by conjugation for 20 of the 43 isolates tested, with a frequency of transfer reaching 1.8 × 10^−3^ (Fig. S8). Most successful donors harbored a representative of plasmid cluster 476, encoding CTX-M-1. Acquisition of the *bla*_CTX-M-1_, *bla*_CMY-2_, and *bla*_TEM-1_ genes by the transconjugants and the replication type of the carrier plasmids (IncI1, IncI2, IncF) were confirmed by PCR in up to three transconjugants per donor (Table 2 and Table S3). For all three donors that harbored *bla*_TEM-1_, the gene was undetectable in the transconjugants, stressing the plasticity associated with the mobility of determinants in their surroundings, which were mainly Tn*2* but also frequently included Tn*3* and 14 other IS elements. All the successful donors were E. coli, with the exception of one Providencia rettgeri isolate. ColRNAI plasmids were transferred concomitantly with IncI1 plasmids as evidenced by sequencing the genome of a transconjugant isolate (Table S3). There was no clear effect of the antibiotic practice on the conjugative potential of the selected isolates.

**TABLE 2:** Primers used in this study

Primer	Sequence (5′–3′)	Target	Amplicon size (bp)	PCR conditions^*a*^	Reference
CMY2-A	TGATGCAGGAGCAGGCTATTCC	CMY-2	323	55°C, 30s	(113)
CMY2-B	CTAACGTCATCGGGGATCTGC				
CTX-U1	ATGTGCAGYACCAGTAARGTKATGGC	CTX-M-1	593	55°C, 30s	(113)
CTX-U2	TGGGTRAARTARGTSACCAGAAYCAGCGG				
MultiTSO-T_for	CATTTCCGTGTCGCCCTTATTC	TEM-1	800	48°C, 50s	(114)
MultiTSO-T_rev	CGTTCATCCATAGTTGCCTGAC				
I1 FW	CGAAAGCCGGACGGCAGAA	IncI1	139	55°C, 30s	(115)
I1 RV	TCGTCGTTCCGCCAAGTTCGT				
RepA-F	CTGTCGGCATGTCTGTCTC	IncI2	533	50°C, 30s	(116)
RepA-R	CTGGCTACCAGTTGCTCTAA				
MRxeF-tot for	ATCAGGAMCCACAGTTACAC	IncFIA, IncFIB, IncFII	753	48°C, 50s	(117)
MRxeF-tot rev	GTTTCATGATRTCRCGACTGAG				
FII FW	CTGATCGTTTAAGGAATTTT	IncFII	258–262	45°C, 30s	(118)
FII RV	CACACCATCCTGCACTTA				
FIA FW	CCATGCTGGTTCTAGAGAAGGTG	IncFIA	462	50°C, 30s	(118)
FIA RV	GTATATCCTTACTGGCTTCCGCAG				
FIB FW	TCTGTTTATTCTTTTACTGTCCAC	IncFIB	683	48°C, 50s	(118)
FIB RV	CTCCCGTCGCTTCAGGGCATT				

a Annealing temperature and elongation time in seconds. All PCR detections were carried out as follows: initial denaturation at 94°C for 2 min, 30 cycles of amplification with denaturation performed at 94°C for 25s, and a final elongation step at 68°C for 5 min. The elongation was performed at 68°C with a different duration for each set of primers.

### Phylogrouping and serotyping of E. coli isolates.

**FIG 5:**
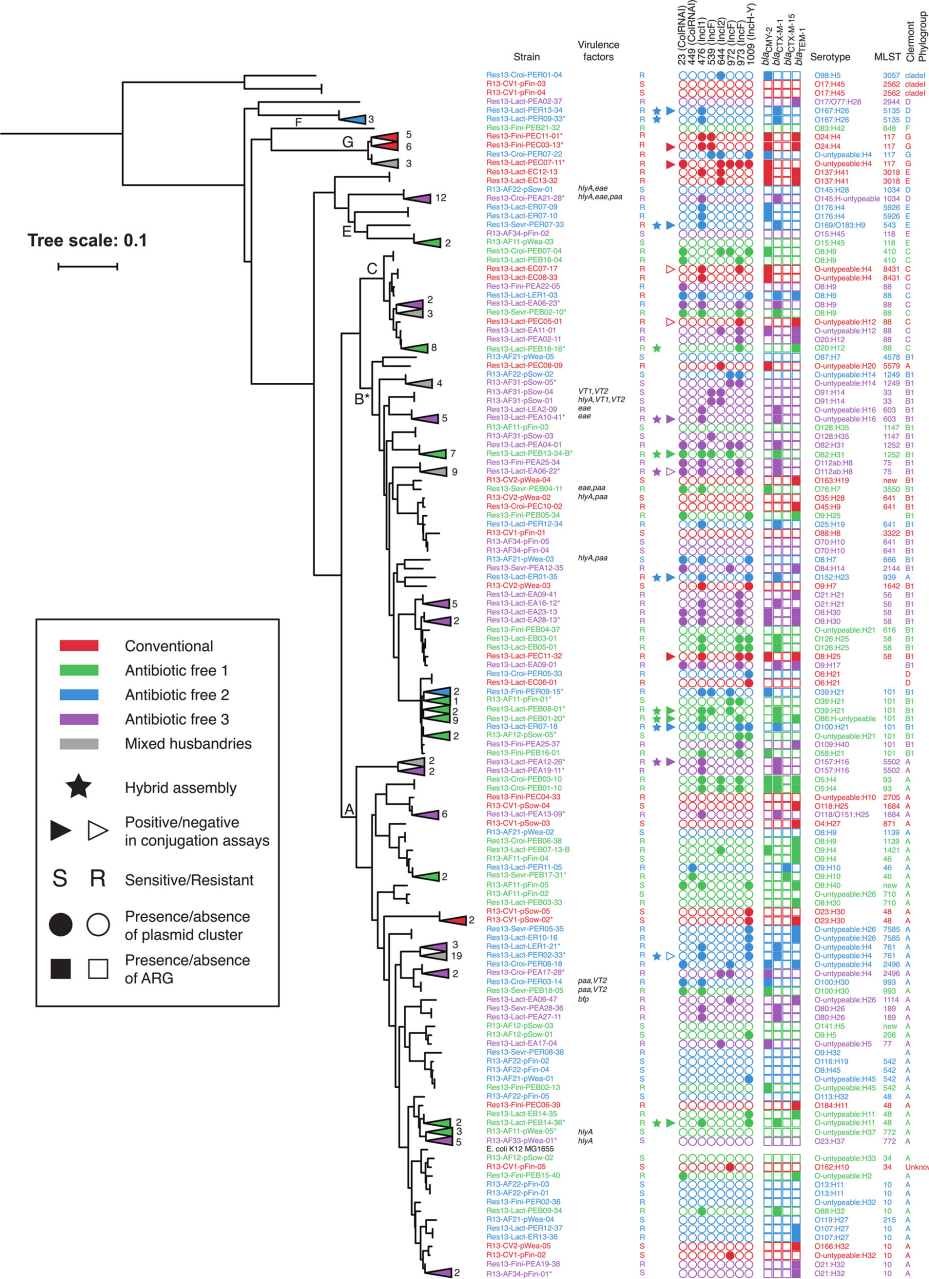
Characterization of E. coli strains. The phylogeny of the porcine E. coli strains susceptible (S) or resistant (R) to CTX in relation to E. coli K-12 was computed using SNVPhyl (24), and graphic representation was generated with iTOL (107). The triangles pointing to the left, and the associated numbers, represent the collapse of individual strains. The strains’ characteristics are represented and defined in the legend.

The single nucleotide variant phylogenomic classification (24), the *in silico* predicted serotypes, the multilocus sequence type (MLST), and the Clermont phylogroups confirmed the polyclonal diversity of the 3GC-resistant E. coli in this study (Fig. 5). The plasmid content and ARG content were also investigated and found to be highly diverse. The E. coli isolates segregated in Clermont phylogroups from which specific lifestyles and hosts can be inferred (25). The A, B1, B2, C, D, E, F, G, and clade I Clermont phylogroups mirrored the SNVPhyl tree branches and were distributed as follows. A and B1 were uncovered in fecal samples from all four husbandries, while C isolates were recovered only in fecal samples from AF1, AF3, and CV.

### Virulence factors of isolates.

Genes encoding virulence factors were found in the set of sequenced CTX-resistant E. coli isolates. The gene *ehxA*, which codes for an enterohemolysin, was always associated with IncF plasmids of cluster 1561 uncovered from AF3-4 group animals. None of the 10 strains carrying *ehxA* showed hemolysin activity under the conditions tested. Genes encoding the porcine attaching and effacing-associated protein (*paa*) were mostly located in the chromosome but were also present on cluster 377 (IncFII, IncFIIA) and were found in AF1, AF2, and AF3 animals. Similarly, the intimin-encoding *eae* genes were mostly associated with the chromosome but were also found on an untyped plasmid. Moreover, *eae* was only encountered in AF1 and AF3 animals. None of the plasmids carrying these virulence factors harbored an ARG. However, *bla*_CTX-M-1_ and *bla*_CMY-2_ were found in isolates also having *ehxA* and/or *eae* and/or *paa* (Fig. 5). The virulence factors were all observed in bacteria isolated form antibiotic-free animals.

## DISCUSSION

### Impact of husbandry on AMR.

The resistome analysis of the sequenced isolates indicate that many genetic determinants of resistance were shared by individuals across the swine production continuum, regardless of age, husbandry, or sequential barn and transport history. This suggests that the microbiota of the sow, which is transferred to offspring, plays a pivotal role in determining the profile of antibiotic-resistant bacteria and ARGs in feces (26). However, differences between husbandries were observed for specific ARGs. Notably, *bla*_CTX-M-1_ was absent from isolates originating from CV settings. CTX-M-1 is widespread, with humans, animals, and the environment considered reservoirs (27). Details of the sows’ origins were not provided by the commercial owners, but it was declared that the CV farm was independent. If it was a closed herd, i.e., a herd in which no animals are imported, the CV animals in our study may simply have not yet acquired enterobacteria harboring *bla*_CTX-M-1_. The absence of *bla*_CTX-M-1_ also indicates that antimicrobial use may not always be associated with presence or absence of an ARG, as the CV herd was receiving penicillin. The resistome of antibiotic-free animals also confirms that ARGs may persist in the absence of the selection pressure posed by antibiotics (28).

Resistance to 3GC was more frequent in antibiotic-free animals, while the bacteria isolated from penicillin-administered animals were on average resistant to a greater number of antibiotics. This suggests that the repeated use of penicillin early after farrowing and after weaning exerted a modulation of the intestinal microbiota favoring a more diverse resistome. In addition, the microbiota populations shared by higher levels of the pig pyramid and environmental factors (e.g., barn and truck microbiomes), which were not assessed in this study, may have been decisive factors in shaping the pig gut *Enterobacteriaceae* populations. The ubiquitous presence of some IncI1 plasmids may at least partly explain the higher frequency of resistance to CTX in antibiotic-free animals.

Most *Enterobacteriaceae* quantified in this study were resistant to TET, which agrees with the commonly observed resistance patterns in swine, cattle, and chickens, the three major Canadian livestock species (29), as well as in humans (30, 31). The ubiquitous prevalence of TET resistance appears to be the outcome of over 70 years of extensive use of this drug in humans and commodity animals (32). TET resistance was less frequent in antibiotic-free animals, in agreement with another study comparing organic and conventional pigs (33). Our results suggest that prophylactic use of penicillin exerts a selective pressure favoring resistance to TET, FOX, STR, TZP, and CHL.

### Effect of animal age on AMR.

As pigs grew older, their TET- and CTX-resistant *Enterobacteriaceae* populations declined. Similar trends were previously noticed in pigs, chickens, and beef cattle (34–37). Furthermore, studies in humans showed higher abundances of ARGs and mobile genetic elements in infants than in adults (26, 38). It was proposed that *Gammaproteobacteria*, and more specifically *Enterobacteriaceae* and E. coli, harbor the majority of the most abundant ARGs in intestinal microbiomes (26, 39). The counts of fecal *Enterobacteriaceae* in the studied pigs decreased with age, which may explain, at least partly, the concomitant reduction in the TET and CTX resistance levels in aging animals. For the CV animals, the duration of time since the antimicrobial was used may also have contributed to the resistance observed. This decrease in resistance abundance with age is in line with the evolution pressure exerted by historic antimicrobial use in suckling and weaning piglets. During pig growth, the phylogenetic composition of the intestinal microbiota changes, and it was shown that the porcine intestinal *Enterobacteriaceae* were overcome by other populations presumably more adapted to the individual intestinal niche (40).

### The resistance determinants.

The results presented agree with existing literature that resistance to β-lactams can be attributed to the concerted actions of low-affinity penicillin-binding proteins, β-lactamases, and efflux pumps (41, 42). Two penicillin-binding proteins emerged as major determinants for resistance to ampicillin and first- and third-generation cephalosporins. These included a variant of PBP3 initially discovered in Haemophilus influenzae (43) described as a pivotal component of the cell division complex (44) and AmpH, which is involved in peptidoglycan recycling (45). Chromosomally encoded AmpC proteins were the first enzymes reported to degrade penicillin (46) and conferred resistance to ampicillin, 1GC, and 3GC. Their expression is low and induced by β-lactam antibiotics and other stimuli (47). AmpC enzymes are located in the bacterial periplasm, and the porins and efflux pumps control the traffic of β-lactams in the outer cell membrane and deliver them to the β-lactamases (48). AmpC are active on penicillins but even more active against 1GC. They can hydrolyze cephamycins, 3GC, 4GC, monobactams, and carbapenem but at a lower rate due to lower affinity (48). Clavulanic acid and tazobactam have a much smaller effect on AmpC β-lactamases, although some isolates are inhibited by tazobactam or sulbactam. In our study, the plasmid-mediated AmpC β-lactamases were CMY-2 and other CMY, MIR-1, and ACT. Like the chromosomal AmpC β-lactamases, the plasmid-mediated enzymes confer resistance to a broad spectrum of β-lactams (48). Interestingly, the ESBL CTX-M-1 was particularly important in a context where CTX was used in combination with clavulanic acid. The only meropenem-resistant isolates recovered were Providencia rettgeri containing an IncQ1 plasmid coding for the metallo-β-lactamase IMP-7; however, no carbapenemase activity was detected. *Providencia* can be intrinsically resistant to carbapenems (21). Nevertheless, IMP-7 often confers carbapenem resistance in *Pseudomonas* (49–51), a genus that colonizes the intestinal mucosa of pigs (52). Even though it was not active in our context, this *bla*_IMP-7_ could be mobilized to a more relevant genetic location and bacterial host and, as such, can be considered a latent threat in the gene flow between bacterial opportunistic pathogens.

### ESBL and coselection.

The portrait of the diversity of ARG arrangements in chromosomes and mobile elements revealed by this study illustrates the successful mediators of 3GC resistance in bacteria of porcine origin. Numerous variants of ARGs and plasmids and their cellular colocalization were observed, stressing the endless genetic diversity driven by genome plasticity. Similar to other studies in animals and humans (53), IncI1 plasmids were the major carrier of the ESBL-encoding genes *bla*_CTX-M-1_ and *bla*_CMY-2_. This study highlights the epidemic nature of IncI1 plasmids carrying *bla*_CTX-M-1_ associated with IS*Ecp*1 in antibiotic-free herds, which agrees with previous work (54). The randomly selected isolates subjected to conjugation assays support these plasmids being primary mediators for the propagation of CTX resistance. In contrast to *bla*_CTX-M-1_, which was only found on IncI1 plasmids, the genes *bla*_CMY-2_, *bla*_CTX-M-15_, and *bla*_TEM-1_ were in a plethora of genetic localizations. The gene coding for the penicillinase TEM-1 was found on the chromosome and on plasmids also carrying ESBL-encoding genes and in close proximity to *cusB* and *cusF*, components of an efflux system conferring resistance to drugs and toxic metals like copper (55). The copper concentrations were high and comparable in pig feeds from all husbandries and may have exerted a selective pressure for the maintenance of *bla*_TEM-1_.

The *bla*_CTX-M-1_, *bla*_CMY-2_, and *bla*_CTX-M-15_ ESBL-encoding genes were all associated with IS*Ec*p1, which has been involved in the mobilization of chromosomal ARGs into plasmids and between plasmids and can provide a strong promoter to neighboring ARGs (48). IS*26* is important in resistance propagation in Gram-negative bacteria and can also provide promoter elements driving the expression of adjacent ARG (56). In our isolates, single copies of IS*26*, probably translocatable units (57), were associated with genes conferring resistance to co-trimoxazole and aminoglycosides on the same CTX-M-1-encoding IncI1 plasmids. Indeed, there is a clear coselection between *bla*_CTX-M-1_, *aadA5*, *dfrA*, and *sul2*, genes conferring resistance to ceftiofur, streptomycin, and co-trimoxazole, antibiotics commonly used as curative treatments in Canadian pig operations (29). Van Gompel et al. (7) previously observed coselection between penicillin, cephalosporins, and amphenicol. The colocalization of *bla*_CTX-M_ and genes conferring resistance to aminoglycosides and fluoroquinolones is also common (58, 59). Moreover, accumulation of genetic determinants mediating non-β-lactam resistance mechanisms are known to contribute to the maintenance of CTX-M-encoding plasmids (60, 61).

### Coexistence of resistance and virulence genes.

Most of the isolates in this study appear to be commensal bacteria, as they did not encode virulence factors. Screening a collection of ∼10,000 isolates initially picked on MAC-CTX exerted a bias toward a highly 3GC-resistant subpopulation of *Enterobacteriaceae*. Among this population, a few strains both were resistant to 3GC and harbored virulence factors, and they were all originating from antibiotic-free husbandries.

Members of the D phylogroup have been shown to cause disease in humans and animals (62) and were recovered from fecal samples of AF2, AF3, and CV but not from AF1 animals. Moreover, phylogroup G strains have proven high virulence and antimicrobial resistance potential (63) and were recovered from both CV and antibiotic-free pigs (Fig. 5). Strains of the seropathotypes O137:H41 Clermont E and O8:H25 Clermont B1 (ST58) were recovered in CV pigs and were previously described as human-only-disease-associated (64) and a serious risk for humans and animals (65).

CTX-M-15-producing enterobacteria have been isolated from humans and animals as well as the environment all over the world (66–69). CTX-M-15 is notably found in E. coli ST131 group B2 (70), but we did not find this configuration in the set of strains studied.

### Conclusion.

The presence of antibiotic-resistant bacteria and ARGs encountered in the porcine intestinal microbiome is the aftermath of many factors, including the dialogue with the pig immune system, the seeding microbiota acquired from the mother, the contamination of feed, water, and the barn and truck environments, husbandry practices, and the exposition to prophylactic and/or therapeutic use of antibiotics in pig productions. The relative contributions of these factors are likely specific to each production context, and their effect on the global AMR crisis remains obscure. In the farms studied, antibiotic-free practices reduced the abundance of fecal bacteria resistant to TET, FOX, STR, TZP, and CHL. Conversely, the ESBL phenotype was encountered much more frequently in bacterial isolates from antibiotic-free pig feces. Notwithstanding, our results indicate that antimicrobial use does not affect the frequency of resistant enterobacteria found on the carcasses. Beyond the impact on resistance frequency, there is a considerable environmental footprint of using large amounts of prophylactic antibiotics that are excreted and make their way into the environment through application of manure on farmland. Overall, our study identified critical resistance determinants and their genetic context, as well as their potential for mobility and coselection with other resistance determinants. Such markers could be used to locate hot spots of AMR transmission from the pig farms to the surrounding environment and could be targeted in evidence-based strategies to reduce antimicrobial resistance in pig production, which may affect both animals and humans.

## MATERIALS AND METHODS

### Animals.

Care of pigs followed the guidelines of the National Farm Animal Care Council (71). All animal procedures were approved by the Institutional Committee on Animal Care of the Sherbrooke Research & Development Centre of Agriculture and Agri-Food Canada according to the guidelines of the Canadian Council on Animal Care (72). This study included samples collected from October 2016 to January 2018 from 78 lactating Yorkshire-Landrace sows and 390 equally distributed male and female piglets conceived with Duroc semen and reared in penicillin-using or antibiotic-free commercial facilities. Pigs were divided into 11 groups of 7 or 8 litters reared with one of the four production practices (Table 1). Two to four groups of animals per husbandry were followed for two to four time points (Table S5). All piglets were provided with creep feed during lactation from ∼7 days of age to weaning at 21 or 28 days of age (Table 1). The feed for piglets up to ∼12 kg contained 2,500 to 3,000 mg/kg of zinc oxide and 29 to 277 mg/kg of copper sulfate, while the concentration of metals was reduced in feed formulated for older animals (100 to 232 mg/kg for zinc and 17 to 173 mg/kg for copper). In CV animals, amoxicillin was administered *per os* to sows during the farrowing period, and all male piglets, approximately half of the piglets studied, received an intramuscular injection of 1 ml of penicillin G procaine at 3 days of age. All pigs were transported between two to three distinct barns separated by ∼10 to 500 km and then were delivered to a single abattoir for processing (Table S5).

### Samples and culture media.

The samples consisted of feeds, feces, and manures of lactating sows collected 14 days after farrowing, swabs from five piglets per sow litter at four growth stages (suckling, weaning, growing, and finishing pigs), and swabs of their carcasses (Table S5). All samples were kept on ice, transported to the Sherbrooke Research and Development Centre within 24 h, and processed upon reception. The feces were collected fresh without contact with the floor, homogenized manually, and kept in sterile Whirl-Pak bags (Nasco, Fort Atkinson, WI, USA). On farm, aliquots were diluted 1:5 into the nonnutritive and osmotically balanced Cary-Blair medium (CB; Dalynn, Calgary, Canada) to preserve viable bacterial cells in anticipation of microbiology analyses. The air was manually removed from the bags containing the remaining samples for molecular biology studies. Samples of feces were received at all sampling time points, while feed and manure samples were sometimes missing. The manure tanks were not always safely accessible. In these cases, manure samples were scooped from the pen floor of pigs raised on slatted flooring or on straw. Manure samples from AF1 and AF2 were homogenized into CB medium prior, as they were solid because of the straw they contained, while manure samples from AF3 and AF4 were liquid and were directly serially diluted into 0.1% peptone water and plated onto selective media.

The procedure used for sampling the carcasses met the requirements of the Hazard Analysis Critical Control Point regulation of the U.S. Food and Drug Administration (73). Briefly, ∼100-kg carcasses were swabbed after 24 h of refrigeration on three 10 cm by 10 cm surface areas (belly, thigh, and jowl) with a sponge moistened with 10 ml of 2% buffered peptone water and stored in Whirl-Pak bags (Nasco) until sampling. The carcass swab sponges were further moistened with 10 ml of 2% buffered peptone water, homogenized using a stomacher for 2 min at normal speed, and then incubated statically for 16 to 18 h at 37°C. The next day, the bacterial suspensions from the sponges were diluted 1:10 in EC broth (MilliporeSigma, Oakville, Canada) supplemented or not with CTX and then incubated for 16 to 18 h at 37°C. Aliquots of the enriched cultures were kept at −80°C in 15% glycerol.

### Bacterial viable counts.

Enterobacteria in feeds, manures, and feces of individual sows and in composite samples of piglets and pigs mixed by litter were enumerated by serial dilution into 0.1% peptone water and then plating on MacConkey II agar (MAC, BD Biosciences, Mississauga, Canada) supplemented or not with cefotaxime (CTX, 2 μg/ml), meropenem (MER, 2 μg/ml), or tetracycline (TET, 8 μg/ml) at half the MIC (21). EC broth supplemented or not with 2 μg/ml CTX was used for enrichment of coliforms in the samples for which few or no colonies were obtained by plating fresh material. The entire feed, feces, and manure sample collection was plated onto MAC, MAC-CTX, and MAC-MER agar plates, while only feces from AF3 and CV growing and finishing animals were used for inoculation of MAC-TET plates due to time limitations and the well-documented fact that TET resistance in porcine enterobacterial populations is highly frequent. The CFU were counted and normalized per gram of wet primary sample.

### Selection of isolates.

To maximize the recovery of ESBL-producing *Enterobacteriaceae*, a minimum of 32 isolated colonies of presumed Escherichia coli originating from feces samples were picked from MAC-CTX and MAC-MER plates and from plates of mSuperCARBA and ESBL media inoculated with serial dilutions of fresh material or streaks of cultures enriched in EC-CTX broth. Isolates were picked from feces of all animal groups, at all ages, except for the CV-1 group at weaning. When obtained, isolates from feed and manure samples were also picked from MAC and MAC-CTX plates. To diversify the media used for isolation of carbapenem- and 3GC-resistant *Enterobacteriaceae* with the aim of favoring a wider diversity of isolates, some samples were also plated into MAC supplemented with half the MIC for CRO (2 μg/ml) or streaked onto mSuperCARBA and ESBL selective media from CHROMagar (Alere, Stittsville, Canada) and HiCrome *Klebsiella* selective agar plates (Himedia, West Chester, PA, USA). Isolates from carcasses were picked from MAC-CRO plates streaked with EC-CTX enriched cultures or from MAC plates for EC enriched cultures. Colonies were picked based on morphology, transferred into 96-well plates containing TSB-CTX or TSB-MER, incubated with agitation for 16 to 18 h at 37°C, and preserved in 15% glycerol at −80°C. Subsets of isolates were purified by streaking twice either on MAC or on Chromocult coliform agar (MilliporeSigma) with the same supplementation as the medium on which the isolate was picked. All inoculated agar plates were incubated statically for 18 to 24 h at 37°C, while broth cultures were incubated with agitation (200 rpm) for 16 to 18 h at 37°C. Isolates were preserved by adding a final concentration of 15% glycerol to overnight cultures in tryptic soy broth (TSB; BD Biosciences) and kept at −80°C. The purified isolates were characterized by various methods (Table S6).

### Pin replicator resistance screening.

A total of ∼10,000 individual colonies originating from the entire sample collection, often with multiple representatives for each sample, was screened by the pin replicator method for resistance to carbapenems, 3GC, and ampicillin as previously described (74) with the following modifications. Briefly, isolates were used to inoculate 96-well plates containing Mueller-Hinton broth (BD, Mississauga, Canada) and statically incubated for 16 to 18 h at 37°C. Culture density was quantified at optical density of 600 nm and diluted with 0.1% peptone water to obtain cell suspensions standardized to about 10^4^ cells per spot (1 μl). These suspensions were spotted with a 96-pin Boekel Scientific replicator (Thermo Fisher Scientific, Waltham, MA, USA) onto a series of Mueller-Hinton agar plates supplemented with CTX, CRO, AMP, TET, MER, and ertapenem (ETP) at 0.5×, 1×, and 2× the MIC as defined by the CLSI guidelines (21). Plates without antibiotics were also inoculated as controls for spot density and used as positive controls for growth.

### Heat maps for visualization of resistance hot spots.

A heat map analysis tool using in-house programming with the SAS software was developed to visualize the data generated by the pin replicator method applied to over 10,000 isolates of resistant enterobacteria in the swine production continuum (75). The resistance observed for CTX, CRO and AMP was consistent between the pin replicator screening and the antibiotic susceptibility testing using microbroth dilutions. Results obtained with the two methods were discordant for TET (33% unmatched) and MER (54% unmatched) and were not considered for the resistance hot spot analysis. The tool was based on the initial number of isolates per sample, the resistance or susceptibility criteria (to CTX and CRO), and the relative dangerousness of the resistance profile obtained. For instance, the resistance to both CTX and CRO is considered more dangerous than the resistance to only one of the two. The resistance or susceptibility criteria were defined as “R” attributed to isolates that grew on 2× the MIC, “J” to isolates that grew on 1× the MIC, and “B” to isolates that grew on 0.5× MIC. The frequency of each profile was calculated, then transformed in percentage of appearance in terms of total number and isolates per sample (litter). The averages for all samples (litters) were then calculated, between 1 and 7 for feces and 1 for feed and manure, and then an indicator analysis of these values was carried out in PC-ORD (76). The potential AMR risk levels for humans of the profiles were defined as weights that were applied to these percentages (weights of 0 to 8, with higher numbers indicating increasing dangerousness). The sum of the profiles for each animal group was calculated and then divided by the total maximum value to obtain a score between 0 and 1, a value of 1 being a highly resistant group.

### Antibiotic susceptibility testing.

A subset of 360 presumptive carbapenem- or 3GC-resistant as well as susceptible isolates were chosen for confirmation of the resistance phenotype by AST for 24 antibiotics using automated broth microdilution and the NARMS Gram-negative CMV4AGNF and extended-spectrum beta-lactamase plates (Sensititre; Thermo Scientific, Waltham, MA, USA), and MIC results were interpreted based on clinical breakpoints according to CLSI M100 (21). Isolates were considered ESBL when the MIC for cefotaxime-clavulanic acid (FTC) or ceftazidime-clavulanic acid (CCV) was at least 3 logs lower than the MIC for CTX or TAZ, respectively. The isolates that were classified carbapenem-resistant by microbroth dilutions profiling were further tested using the mCIM (77) to confirm the production and secretion of active carbapenemases.

### Hemolytic activity testing.

The hemolytic activity of isolates with genes putatively coding for hemolysins was assessed by streaking on Columbia blood agar with 5% sheep blood (Thermo Scientific, Nepean, Canada). Plates were incubated statically up to 48 h at 37°C.

### Preparation of bacterial genomic DNA, libraries, and sequencing.

A selection of 60 susceptible and 244 3GC-resistant bacterial isolates was cultured for 3 to 6 h at 37°C in Brain-Heart Infusion broth (Oxoid Ltd., Ottawa, Canada) supplemented with 2 μg/ml CTX when required for the maintenance of the resistance phenotype. Genomic DNA (gDNA) was purified from 200-μl aliquots of culture using the Maxwell 16 Cell SEV DNA purification kit (Promega, Madison, WI) according to the manufacturer’s instructions. Final elution was done in 100 μl of elution buffer. gDNA was quantified using the Quant-it High-Sensitivity DNA assay kit (Life Technologies Inc., Burlington, Canada). Sequencing libraries were constructed from 1 ng of gDNA using the Nextera XT DNA Sample Preparation kit and the Nextera XT Index kit as recommended by the manufacturer (Illumina Inc., Vancouver, Canada). Genomic sequencing was performed on the Illumina MiSeq Platform with a 600-cycle MiSeq reagent kit v3 (Illumina Inc.). The sequencing technical data are available in Table S7.

### CFIA-OLC workflow for bacterial assembly and typing (COWBAT).

The COWBAT workflow consists of three major components (quality assessment/quality control, assembly, and typing) and can be found online at https://github.com/OLC-Bioinformatics/COWBAT (78).

### (i) Quality assessment/quality control.

The quality of raw reads was assessed with FastQC version 0.11.8 (79). Adapter removal and quality trimming was performed with bbduk.sh from the BBTools suite version 38.22 (80) with the following parameters: trim quality of 10 and removal of reads below 50 bp long. Error correction was performed using Tadpole version 8.22 (80) in “correct” mode with default parameters. Sequences were screened for potential contamination with ConFindr 0.4.7 (81).

### (ii) Assembly.

Genomic assemblies were conducted with SKESA version 2.3.0, with the vector percent argument disabled (82). One round of automatic assembly improvement was then performed with Pilon version 1.22 (83). Metrics were calculated with an in-house Python script and Qualimap version 2.2.2 (84). The numbers and sizes of open reading frames (ORFs) were determined with Prodigal version 2.6.3 (85). Raw reads and contigs were classified to the genus level with CLARK version 1.2.5 (86).

### (iii) Typing.

MASH version 2.0 (87) was used to compute the distance of the assembled genome to all the genomes present in the RefSeq bacterial database. Ribosomal multilocus sequence typing (rMLST) and 16S rRNA typing were performed on the raw reads against databases downloaded from http://pubmlst.org/rmlst/ (88, 89) and https://ftp.ncbi.nlm.nih.gov/blast/db/, respectively. Typing with raw reads was performed with a reference mapping-based Python script adapted from Lambert et al. (90). Briefly, reads with identity to sequences in the database were “baited” using bbduk.sh version 38.22 and used to subsequently bait sequences from the database. The baited reads were mapped to the baited database sequences with bowtie2 version 2.3.4 (91) and parsed using sipprverse (92). Among targets searched for were pathogen-specific targets (“GeneSippr”) and genomically dispersed conserved sequence (GDCS) probes derived from rMLST genes, as well as antimicrobial resistance genes, virulence genes, prophages, and genes involved in Escherichia coli serotype determination. Databases were downloaded from the Centre for Genomic Epidemiology repository (https://bitbucket.org/genomicepidemiology/) (93, 94). The virulence genes were detected by performing a search of reference sequences against a custom NCBI BLAST+ protein database consisting of coding DNA sequences (CDS) from genomes of all strains included in this paper. Preparation of the strain database and parsing of the blastp outputs have been done with BioPython and are documented in Jupyter Notebook (95). To detect genes coding for an enterohemolysin (*hlyA*) and a porcine attaching and effacing-associated protein (*paa*), the NCBI reference sequence WP_011310119 and GenBank sequence U82533.4 were used, respectively. Similarly, CDS from *bfp* genes encoded on Escherichia coli strain B171 plasmid pB171 described under GenBank accession number AB024946 (96) were used as distinct queries to screen for the *bfp* genes. Finally, 143 complete *eae* sequences were clustered with CD-HIT in 11 divergent sequences showing no more than 90% similarity, and the latter sequences were used as queries to detect intimin genes.

### Long-read sequencing and hybrid assemblies.

In addition, a subset of 40 isolates were subjected to long-read sequencing (Oxford Nanopore Technologies, Oxford, UK) to increase their resolution by hybrid assembly of short and long reads, reducing the average number of contigs per genome from 122 down to 11. As detailed in Table S7, this operation allowed the recovery of 28 complete bacterial chromosomes and 108 complete plasmid sequences. Genomic DNA was extracted by phenol-chloroform (97) or with Genomic-tip 500/G columns (Qiagen). Long-read DNA libraries were prepared with Rapid Sequencing kits SQK-RAD004 and SQK-RBK004 from Oxford Nanopore Technologies (ONT). A total of 10 sequencing runs on ONT’s MinION sequencer with 10 flow cells (ONT FLO-MIN106.1) were performed. The first run was done on a single isolate, while the other runs were multiplexed using 5 to 12 barcodes per run. An average of >32,000-bp reads per isolate was obtained (Table S7). The flow cells were carefully washed between runs according to the manufacturer’s recommendations (ONT EXP-WSH002). Demultiplexing was performed with the Albacore base calling software (98). The read_fast5_basecaller.py command was used with the following options: –flowcell FLO-MIN106 –recursive –kit SQK-RBK004 –barcoding –output_format fastq –worker_threads 12. When required, demultiplexed reads from distinct runs belonging to the same isolate were combined in a single fastq file. Filtlong version 0.2.0 was used to filter the MinION long reads according to length and read identity relative to Illumina paired-end read references (99). The following command was used: filtlong −1 short_reads/illumina_R1.fastq.gz −2 short_reads/illumina_R2.fastq.gz –min_length 1000 –keep_percent 90 –target_bases 500000000 long_reads/raw_nanopore.fastq.gz | gzip > filtered_nanopore.fastq.gz. High-quality hybrid assemblies were recovered for a subset of 40 strains that were sequenced with both Nanopore and Illumina MiSeq sequencing technologies (Table S7). Briefly, Unicycler version 0.4.7 (100) was used in the default conservative mode with paired sequence data sets from Illumina MiSeq reads and Filtlong-filtered Nanopore long reads. The following options were used with the Unicycler command: −1 R1.fastq.gz −2 R2.fastq.gz −l filtlong_filtered_nanopore.fastq.gz -t 16 –keep 2 –verbosity 1 –spades_tmp_dir spaTMP.

### Genome annotation.

Assemblies were annotated using Prokka version 1.13.3 (101) using the CARD protein homolog model database as the primary source for annotation (102, 103). For a refined annotation of plasmids, a custom database with representative sequences of the following ARG-containing plasmid clusters as classified by MOB-suite was used: 473 (JN983049), 476 (CP016522, KJ484637), 644 (JN983044), 659 (KX434884), 972 (JN983046), 973 (CP000836, CP016042), 1009 (CP016549), and 2087 (NC_011513). Briefly, we used the prokka-GenBank_to_fasta_db perl script included in Prokka to produce a Prokka-compatible protein sequence fasta file. To remove redundancy, CD-HIT version 4.6 was used with the following parameters: -T 0 -M 0 -g 1 -s 0.8 -c 0.90 (104, 105). The output file of CD-HIT was renamed PLASMIDS and copied in the prokka/db/Kingdom/Bacteria Prokka installation folder. To ensure that this custom database was used by the Prokka engine, the prokka –setupdb was run and the Prokka executable file was slightly edited to allow similarity searches in the PLASMIDS database. Finally, the following Prokka options were used: –force –addgenes –genus –species –strain –proteins card_protein_homolog_model.fasta –evalue 1e−09.

The isolates carrying resistance genes potentially conferring resistance to carbapenems were further tested using the mCIM (77) to confirm or disprove the production and secretion of active carbapenemases.

### Identification of plasmids, detection of ARG, and their genetic context.

MOB-recon from the MOB-suite version 2.0.0 was used to reconstruct and identify sequences belonging to plasmids (23). For each isolate, the chromosomes and plasmid sequences were screened for the presence of ARG using the Comprehensive Antibiotic Resistance Database (CARD) version 3.03 and the companion Resistance Gene Identifier (RGI) software version 4.2.2 (102). Custom Perl and Python scripts were used to parse the several output files produced by RGI and MOB-suite. The GENcontext tool (106), developed by our group, was used on either hybrid or short-read assemblies to uncover the proximal and long-range genomic contexts surrounding *bla*_CTX-M-1_, *bla*_CMY-2_, *bla*_CTX-M-15_, and *bla*_IMP-7_.

### Phylogrouping.

A refined phylogenetic classification of 247 Escherichia coli strains was performed with a Galaxy-hosted SNVPhyl (24) workflow edited to remove the dinucleotide filtering step. The E. coli strain K-12 substrain MG1655 complete genome (NC_000913.3) was used as a reference. The workflow was used with the default settings with the exception of the following input parameters: a minimum coverage of 15, a minimum mean mapping of 30, and a single nucleotide variant (SNV) abundance ratio of 0.75. The output phylogenetic tree was rooted at the midpoint node and visualized with the Interactive Tree of Life v4 (107). The short-read assemblies of the 247 E. coli and 6 Escherichia fergusonii isolates included in this study were subjected to a local instance of ClermonTyping (25) with the contig length cutoff value set to 2,000 bp.

### Bacterial conjugation.

Conjugation assays were performed as described by Burrus and Waldor (108) with the exception that donor and recipient cells were harvested from broth cultures by centrifugation separately and then mixed together before plating onto LB agar without supplementation, and E. coli strain CV601 (KAN^R^, RIF^R^) was used as the recipient. Transconjugants were selected by plating the resuspended mated cells onto LB agar containing 50 μg/ml kanamycin (KAN), 50 μg/ml rifampin (RIF), and 2 μg/ml CTX. The isolates were then purified by consecutive streakings on the same supplemented medium. Detection of the plasmid replicase type and *bla*_CTX-M-1_, *bla*_CMY-2_, and *bla*_TEM-1_ was performed by PCR using specific primers and reaction conditions indicated in Table 2 and the OneTaq Quick-Load 2× Master Mix with Standard Buffer according to the manufacturer’s instructions (New England BioLabs, Whitby, ON, Canada).

### Statistics.

The phenotypic and genotypic clustering and nonmetric multidimensional scaling (NMS) were computed using PC-ORD 6.0 and based on Bray-Curtis distances (76). To generate genotype-phenotype associations, the genomes of isolates subjected to both AST and sequencing were used for concordance assessments between the presence of genes potentially conferring resistance and the resistance phenotype observed by microbroth dilutions. The genotype-phenotype associations were done on 174 Escherichia coli, 2 *E. fergusonii*, 1 Escherichia hermannii, 7 Enterobacter cloacae, 4 Providencia rettgeri, and 2 Citrobacter freundii isolates obtained from fecal-manure samples from sow, suckling, weaning, growing, and finishing pigs or carcass swabs representing all four husbandry practices. A custom R script was developed to perform the genotype-phenotype associations by using a phenotype data set, a genotype data set, and a resistance determinant data set (109) (Table S1). For each phenotype of resistance to an antibiotic (e.g., CTX), determinants suspected to be implicated in the resistance were retained. The lists of resistance-conferring candidate genes included genes associated with the antibiotic class of the antibiotics tested as defined by the CARD database (102). Genes localized on chromosomes and plasmids were included, and all the genes encoding subunits of functional complexes (e.g., efflux pumps) were required in the same genome for the complex to be considered “present” (Table S1). Then, for each determinant, the number of strains that harbor the determinant (freq_presence) or not (freq_absence) were summed and two values were obtained: the number of strains that harbor the resistance gene and present the observed resistance (var11) and the number of strains that lack the resistance gene and do not present the observed resistance (var00). Finally, the concordance between the presence of a specific determinant and the observed resistance (pct_11) and the concordance between the absence of a specific determinant and the absence of the observed resistance (pct_00) were, respectively, calculated by dividing var11 by freq_presence and var00 by freq_absence. A correspondence analysis was done using the CORRESP procedure in SAS on a matrix generated with the 27 plasmid clusters found in fecal samples (75). The MOB-suite typer tool categorizes the unclassifiable plasmids into “novel” clusters. These clusters are generated for each strain independently and named according to the order of encounter. Novel plasmids were excluded from this correspondence analysis to eliminate the noise caused by the diversity of plasmids that can be encountered in random orders by the MOB-suite typer when sequentially analyzing hundreds of genomes.

### Data availability.

The collection of characterized 3GC-resistant and susceptible porcine isolates of *Enterobacteriaceae* and Acinetobacter species is part of a Canadian legacy collection included in the Integrated Rapid Infectious Disease Analysis Project (110). Genome sequences have been deposited in the NCBI BioProject database under accession number PRJNA662792 (111).

## Supplementary Material

Supplemental file 1

Supplemental file 2

Supplemental file 3

Supplemental file 4

Supplemental file 5
