# Supplementary material for: Resistance Determinants and Their Genetic Context in Enterobacteria from a Longitudinal Study of Pigs Reared under Various Husbandry Conditions
Source: Appl Environ Microbiol. 2021 Mar 26;87(8):e02612-20. doi: 10.1128/AEM.02612-20 (PMC8091121; doi:10.1128/AEM.02612-20)
Supplement: Supplemental file 1 [file AEM.02612-20-s0001.pdf]

Appendixes for the following article published in the

Applied and Environmental Microbiology Journal

Title: Resistance determinants and their genetic context in enterobacteria from a longitudinal study of pigs reared under various husbandry conditions

Running title: Resistance genes and their context in pig production

Authors and Affiliations: Dominic Poulin-Laprade[1#], Jean-Simon Brouard[1], Nathalie Gagnon[1], Annie Turcotte[1], Alexandra Langlois[1], J. Jacques Matte[1], Catherine D. Carrillo[2], Rahat Zaheer[3], Tim McAllister[3], Edward Topp[4], Guylaine Talbot[1]

[1]Sherbrooke Research and Development Centre, Agriculture and Agri-Food Canada

[2]Ottawa Research and Development Centre, Canadian Food Inspection Agency

[3]Lethbridge Research and Development Centre, Agriculture and Agri-Food Canada

[4]London Research and Development Centre, Agriculture and Agri-Food Canada

# Corresponding author:

2000 College Street, Sherbrooke, QC, Canada, J1M 1Z7

Phone: 1-819-571-0864

E-mail: Dominic.Poulin-Laprade@canada.ca

Abbreviations

AMR: antimicrobial resistance
ARG: antibiotic resistance genes
3GC: third generation cephalosporin
ESBL: extended-spectrum beta-lactamase
CARD: Comprehensive Antibiotic Resistance Database
RGI: Resistance Gene Identifier
AF1: organic or antibiotic-free 1
AF2: certified-humane or antibiotic-free 2
AF3: AGRO-COM or antibiotic-free 3
CV: conventional
CB: Cary-Blair medium
MAC: MacConkey II agar
TSB: Tryptic soy broth
EC: *Escherichia coli* broth
0.1% PW: 0.1% peptone water
2% BPW: 2% buffered peptone water
CFU: Colony forming unit(s)
AMP: ampicillin
AUG: augmentin (amoxicillin-clavulanic acid)
TZP: piperacillin-tazobactam
FAZ: cefazolin
CEP: cephalothin
CTX: cefotaxime
POD: cefpodoxime
TAZ: ceftazidime
CRO: ceftriaxone
FTC: cefotaxime-clavulanic acid
CCV: ceftazidime-clavulanic acid

FEP: cefepime
FOX: cefoxitin
IMP: imipenem
MER: meropenem
ETP: ertapenem
SUL: sulfisoxazole
SXT: trimethoprim-sulfamethoxazole
TET: tetracycline
GEN: gentamicin
STR: streptomycin
AZI: azithromycin
CIP: ciprofloxacin
NAL: nalidixic acid
CHL: chloramphenicol

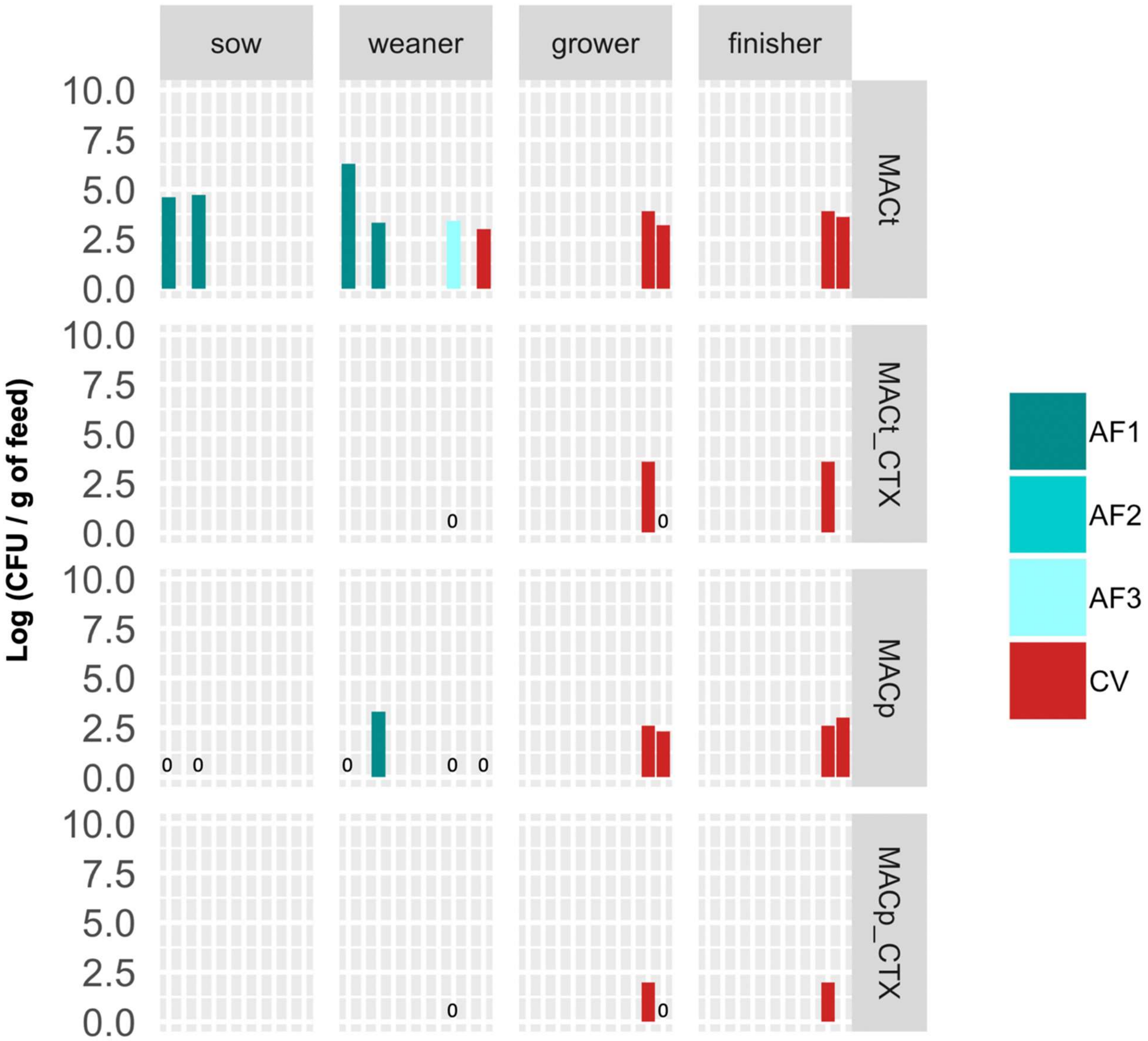

FIG S1 Antibiotic resistance levels in feed. Bacterial counts of pink (MACp) or total (MACt; pink + white) colony forming units (CFU) per gram of feed sample. The bars represent the log transformations of CFU on MacConkey II agar plates supplemented or not with cefotaxime (MACt_CTX, MACr_CTX). Plates devoid of CFU are indicated by a 0, while the missing data is due to missing feed samples.

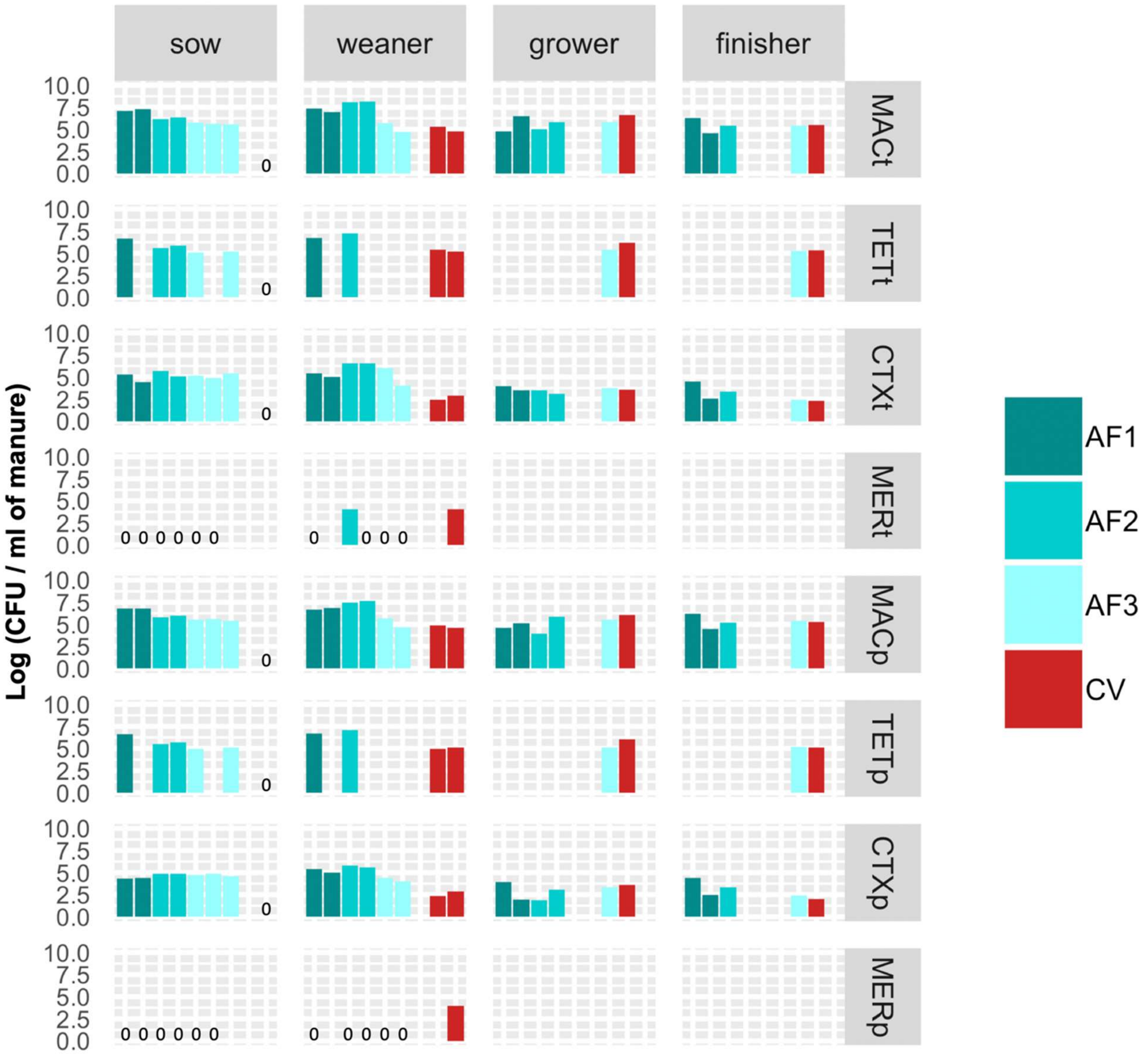

FIG S2 Antibiotic resistance levels in manure. Bacterial counts of pink (p) or total (t; pink + white) colony forming units (CFU) per milliliters of manure sample. The bars represent the log transformations of CFU obtained for each animal group on MacConkey II agar plates without supplementation (MACt, MACp) or supplemented with tetracycline (TETt, TETp), cefotaxime (CTXt, CTXp) or meropenem (MERt, MERp). No CFU counts are indicated by a 0, while the missing data is due to missing manure samples.

sample_type=feces

animal_type	AF3-3	AF3-4	CV1	CV2	AF1-1	AF1-3	AF2-1	AF2-2
sow	0.798	0.873	0.346	0.526	0.995	0.547	0.978	0.896
suckler	0.855	0.921	0.333	0.451	0.995	0.899	0.986	0.901
weaner	0.747	0.767	0.000	0.143	0.916	1.000	0.921	0.910
grower	0.830	0.759	0.595	0.305	0.915	0.875	0.789	0.850
finisher	0.811	0.824	0.719	0.995	0.837	0.735	0.975	0.935

sample_type=feed

animal_type	AF3-3	AF3-4	CV1	CV2	AF1-1	AF1-3	AF2-1	AF2-2
sow	.	.	0.000	.	0.738	0.000	0.083	0.422
weaner	0.713	.	0.000	0.848	0.692	0.675	.	0.286
grower	.	.	0.000	0.292	0.556	0.625	.	.
finisher	.	.	0.900	0.933	1.000	1.000	.	.

sample_type=manure

animal_type	AF3-3_4	CV-1	CV-2	AF1-1	AF1-3	AF2-1	AF2-2
sow	0.615	.	0.663	1.000	0.942	0.981	0.846
weaner	.	0.333	0.869	0.902	0.925	0.877	0.955
grower	1.000	0.870	.	0.884	0.858	0.780	0.718
finisher	0.800	0.500	.	0.842	0.667	0.929	.

FIG S3 Conventional animals carry fewer cephalosporin-resistant *Enterobacteriaceae* than antibiotic-free animals based on the ~10 000 isolates collection. Heat map showing hotspots of CTX and CRO resistant isolates in the experimental set-up. The colour code is as follows: the more resistant, the redder (values between 0.51 and 1); grey, no change (value of 0.5); the less resistant, the greener (values between 0 and 0.49). Dots represent missing data.

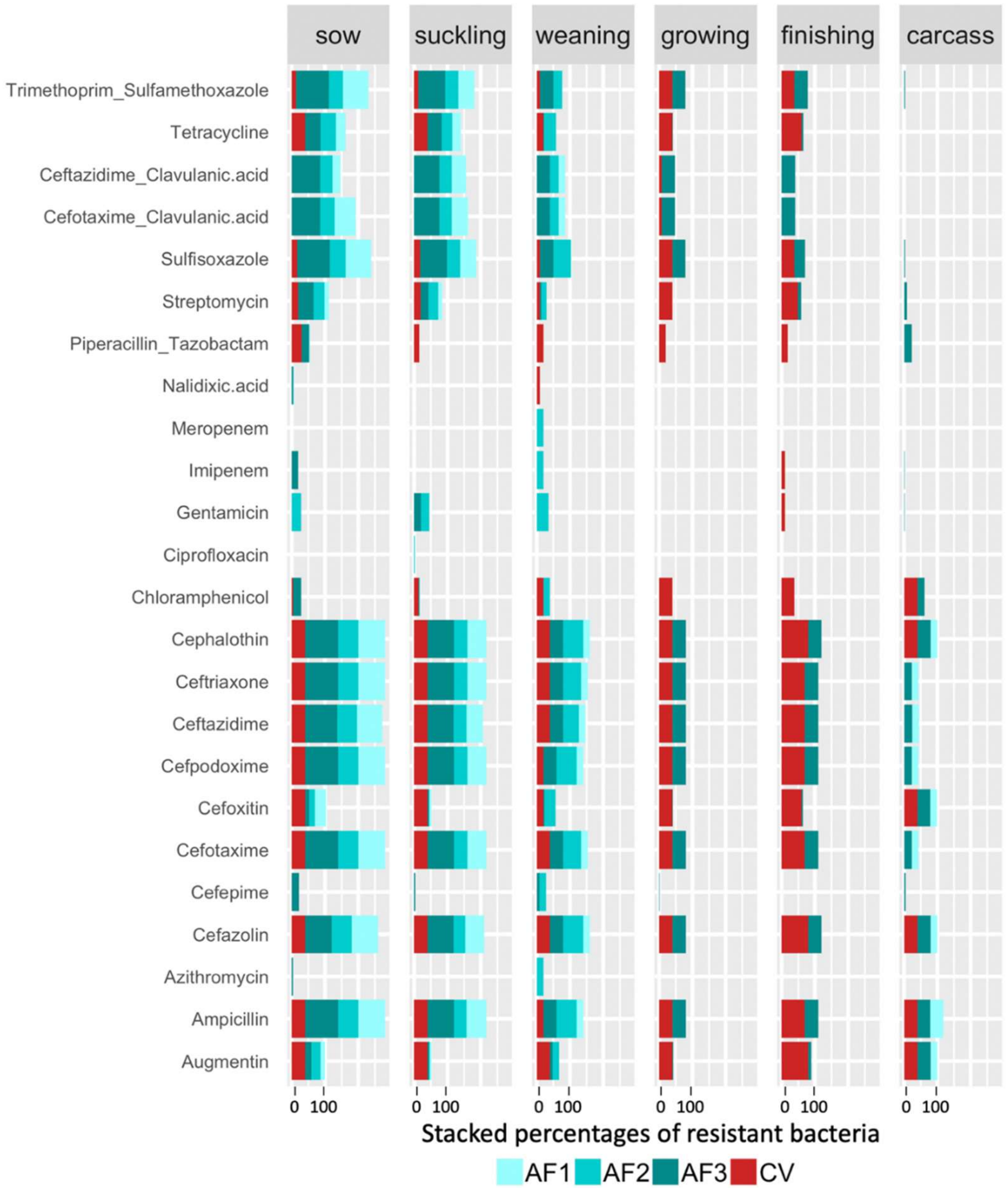

FIG S4 Antibiotic susceptibility testing of porcine CTX-resistant isolates. Antibiotic susceptibility testing on 300 isolates resistant to CTX from feces, manures and carcass swabs for 24 antibiotics using automated microbroth dilution. MIC results were interpreted based on clinical breakpoints according to CLSI M100 (1). The resistant, intermediate, and considered ESBL phenotypic results were converted to 1, and susceptible, dose dependent and no interpretation results were converted to 0. The bars represent stacked percentages of means for the four husbandries.

TABLE S1. Methodology and results associated with the phenotype-genotype concordance calculations. See joined Excel spreadsheet TableS1.

TABLE S2. Detailed report of carbapenemase activity using the mCIM method. See joined Excel spreadsheet TableS2.

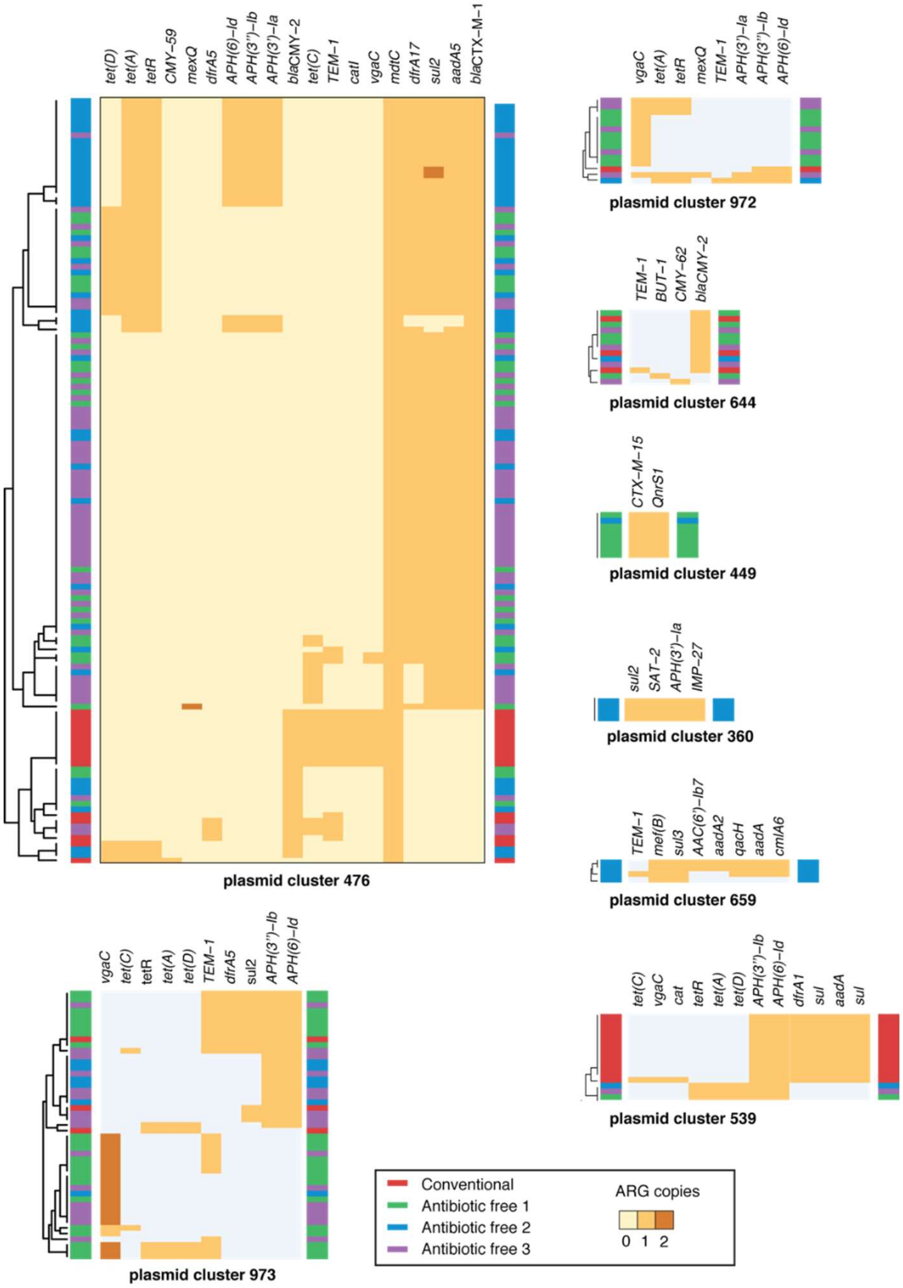

FIG S5 Heat maps of ARG located on the main ARG-encoding plasmid clusters. The ARG names are indicated on top, while the husbandry group of each isolate is indicated by colours on both sides of the heat map. The heatmap.2 function from the ggplot2 R package with the default clustering method (complete linkage method with Euclidean distance measure) was used for clustering data and producing heat maps.

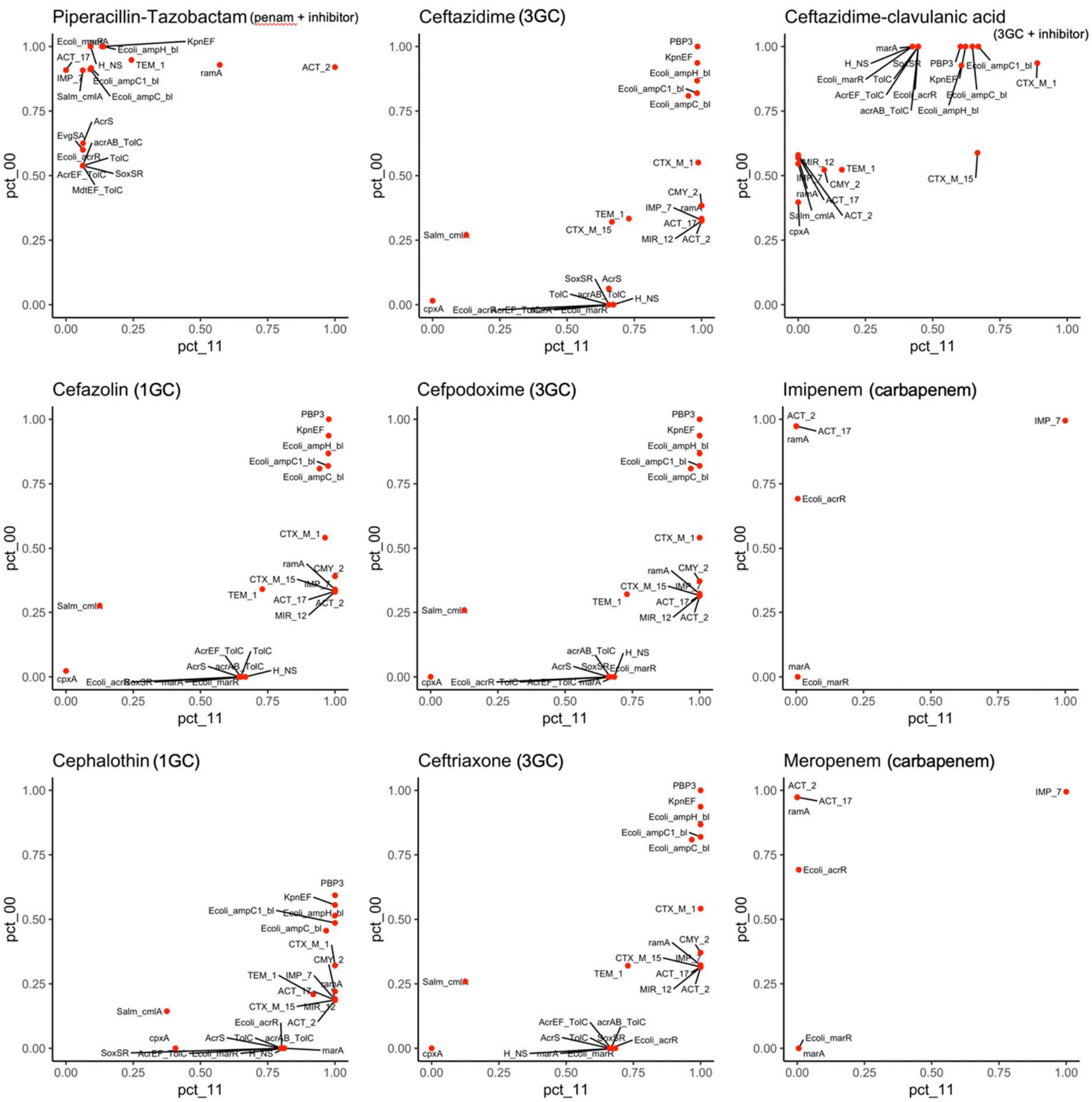

FIG S6 Concordance between the resistome and the resistance observed by antibiotic susceptibility testing of isolates. The dots represent individual genes and their position is at the intersection between the percentage of susceptible isolates without the gene (pct_00) and the percentage of resistant isolates with the gene (pct_11). The antibiotics tested and the associated antibiotic classes are indicated on top of the graphs. Detailed descriptions of the genes are located in Table S1 (continued on the next page).

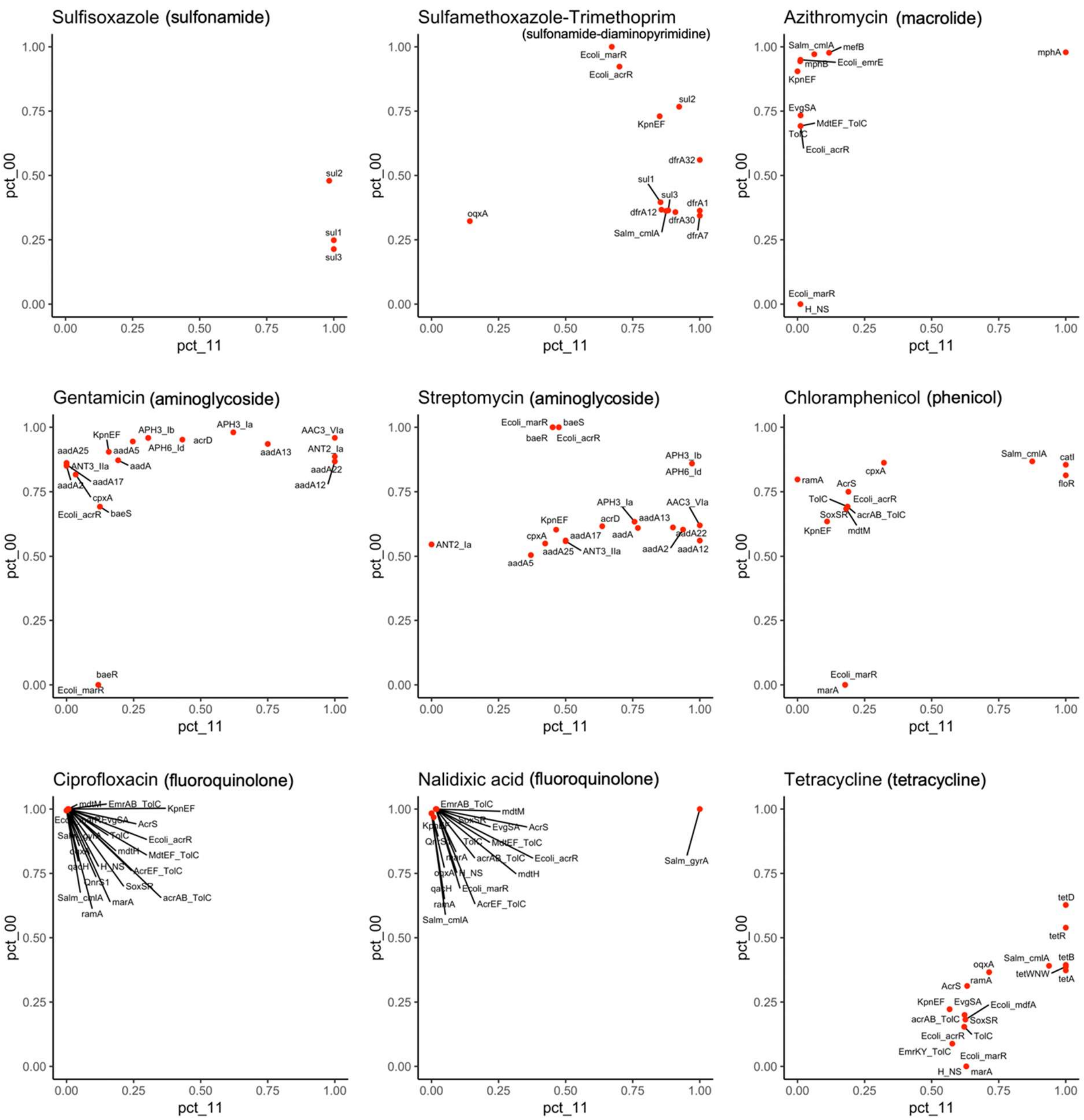

FIG S6 (suite) Concordance between the resistome and the resistance observed by antibiotic susceptibility testing of isolates. The dots represent individual genes and their position is at the intersection between the percentage of susceptible isolates without the gene (pct_00) and the percentage of resistant isolates with the gene (pct_11). The antibiotics tested and the associated antibiotic classes are indicated on top of the graphs. Detailed descriptions of the genes are located in the Table S1.

TABLE S3. Detailed report of the plasmids in the studied bacterial isolates and transconjugants.

See joined Excel spreadsheet TableS3.

TABLE S4 Plasmid groups identified in this study (2).

Rep_type	MobSuite plasmid clusters	n	Median length (bp)	Relaxase type(s)	Mpf type	Predicted mobility[1]	AMR genes[2]
IncA/C2	960, 966	2	146,622	MOBH	MPF_F	C	
IncI1	476	129	113,296	MOBP	MPF_I	C	*aadA5*, APH(3'')-Ib, APH(3')-Ia, APH(6)-Id, CMY-2, CTX-M-1, TEM-1, *catI*, *tet*(A), *tet*(C), *tet*(D), *tetR*, *mdtC*, *mexQ*, *sul2*, *dfrA17*
IncI1	473, 487	5	90,277	MOBP	MPF_I	C, N	*aadA*, *sul1*
IncI2	644, novel	18	63,833	MOBP	MPF_T	C, N	BUT-1, CMY-2
IncN	934	5	45,990	MOBF	MPF_T	C, N	
IncQ1	361	2	4,967	-	-	N	
IncX4	585, 586	2	33,875	MOBP	MPF_T	C	
Inc13	839	6	2,113	-	-	N	
IncFIB, IncFIA, IncFII, IncFIIA, IncY	972	16	95,196	MOBF, MOBP	MPF_F	C, M, N	*vgaC*, APH(3'')-Ib, APH(3')-Ia, APH(6)-Id, TEM-1, *tet*(A), *tetR*
IncFIA, IncFIB, IncFII, IncFIIA, IncQ1	973	52	144,160	MOBF, MOBP	MPF_F	C, M, N	*vgaC*, APH(3'')-Ib, APH(3')-Ia, APH(6)-Id, TEM-1, *tet*(A), *tet*(D), *tetR*, *sul2*, *dfrA5*
IncFII, IncFIIA, IncQ1, IncFIB	539	24	104,493	MOBF, MOBP	MPF_F	C, N	*aadA*, APH(3'')-Ib, APH(6)-Id, *tet*(A), *tet*(D), *tetR*
IncFIA	novel	13	14,172	-	-	N	
IncFIIA	489	1	88,588	MOBP	MPF_I	C	CMY-2
IncFIB	87, 539, 553, 969, novel	48	12,427	-	-	N, M	
IncR	729, 730, 731, novel	14	27,761	MOBF, MOBP	MPF_T	C, M, N	APH(3'')-Ib, APH(6)-Id, *tet*(A), *tet*(D), *tetR*
IncH, IncY	1009, novel	45	83,694	-	-	Non-mobilizable	AAC(3)-Vi, *aaadA*, *sul1*, *sul2*, *dfrA17*, *vgaC*

TABLE S4 Plasmids groups identified in this study (continued).

Rep_type	MobSuite plasmid clusters	n	Median length (bp)	Relaxase type(s)	Mpf type	Predicted mobility[1]	AMR genes
IncX1	39, 46, 47, 55, 55, 825, novel	68	37,904	MOBP	MPF_T	C, N	-
ColRNAI rep cluster 1291	229, 509, 834	27	5,463	MOBQ		M	-
ColRNAI rep cluster 1857	14, 17, 18, 19, 22, 23, 26, 29, 32, 33, 34, 35, 259, 292, 305, 1071	176	4,604	MOBF, MOBP	-	M	-
ColRNAI rep cluster 1885	414, 424, novel	37	1,549	-	-	M, N	-
ColRNAI rep cluster 1987	3, 5, 106, 132, 155, novel	24	5,169	MOBF, MOBP	-	M	-
ColRNAI rep cluster 1993	novel	1	1,943	-	-	N	-
Rep cluster 48	novel	2	244,618	-	-	N	-
Rep cluster 312	497	1	27,727	MOBP	MPF_T	C	-
Rep cluster 488	920	22	108,882	-	-	N	-
Rep cluster 1527	novel	1	45,271	-	-	N	-
Rep cluster 1778	465, 471	16	4,067	MOBQ	-	M	-
Rep cluster 1785	469	2	4,073	MOBQ	-	M	-
-	2087	2	9,496	-	-	N	APH(3'')-Ib, APH(3')-Ia, APH(6)-Id, CMY-2
-	449	7	16,977	-	-	N	CTX-M-15, *qnrS1*
-	659	4	9,418	-	-	N	AAC(6')-Ib7, *aadA*, *aadA2*, *cmlA6*, *mefB*, *qacH*, *sul3*
	1068	55	13,224	-	-	N	-
IncFIIA,IncFII,IncFIB	1561	18	67,128	MOBF			-
-	2911, 2912	29	25,170	-	-	N	-
-	540, 541, 542	19	51,956	MOBF, MOBP	MPF_F	C, N	-

[1] C: Conjugative, N: Non-mobilisable, M: Mobilisable

[2] The antibiotic resistance genes identified by RGI (3) and harbored in the plasmid groups

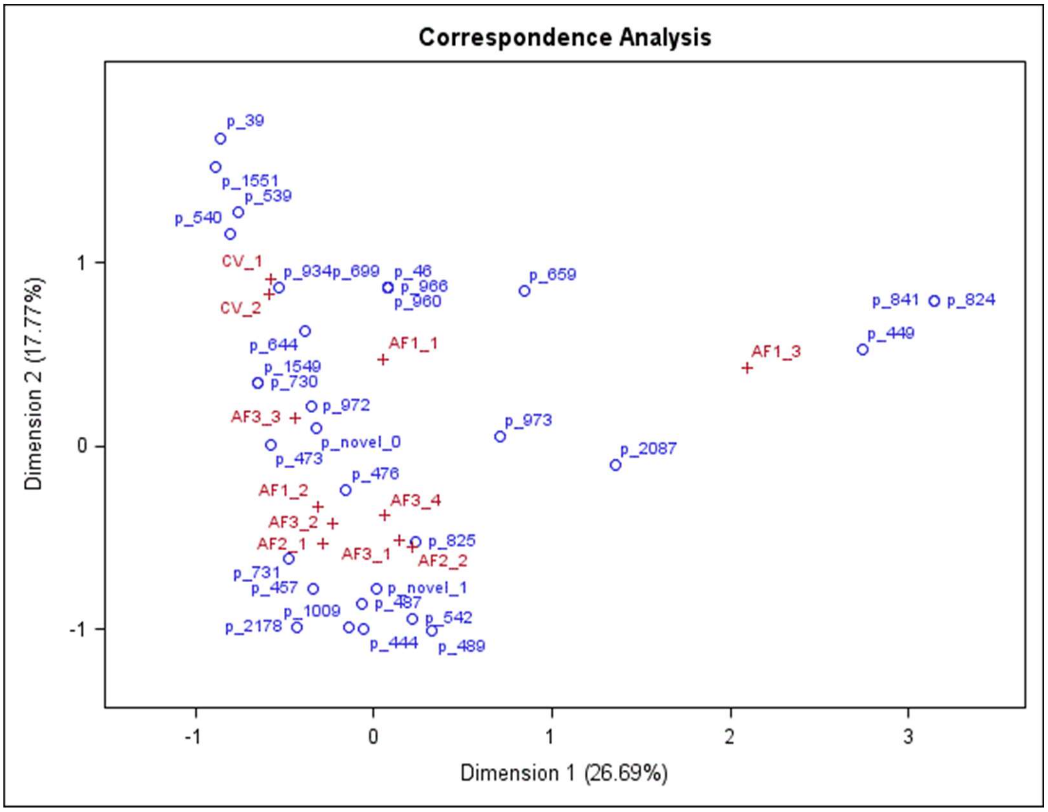

FIG S7 Plasmids and ARG. A) Correspondence analysis on the matrix of 27 plasmids found in feces samples. Circles represent plasmid clusters while plus signs indicate animal groups.

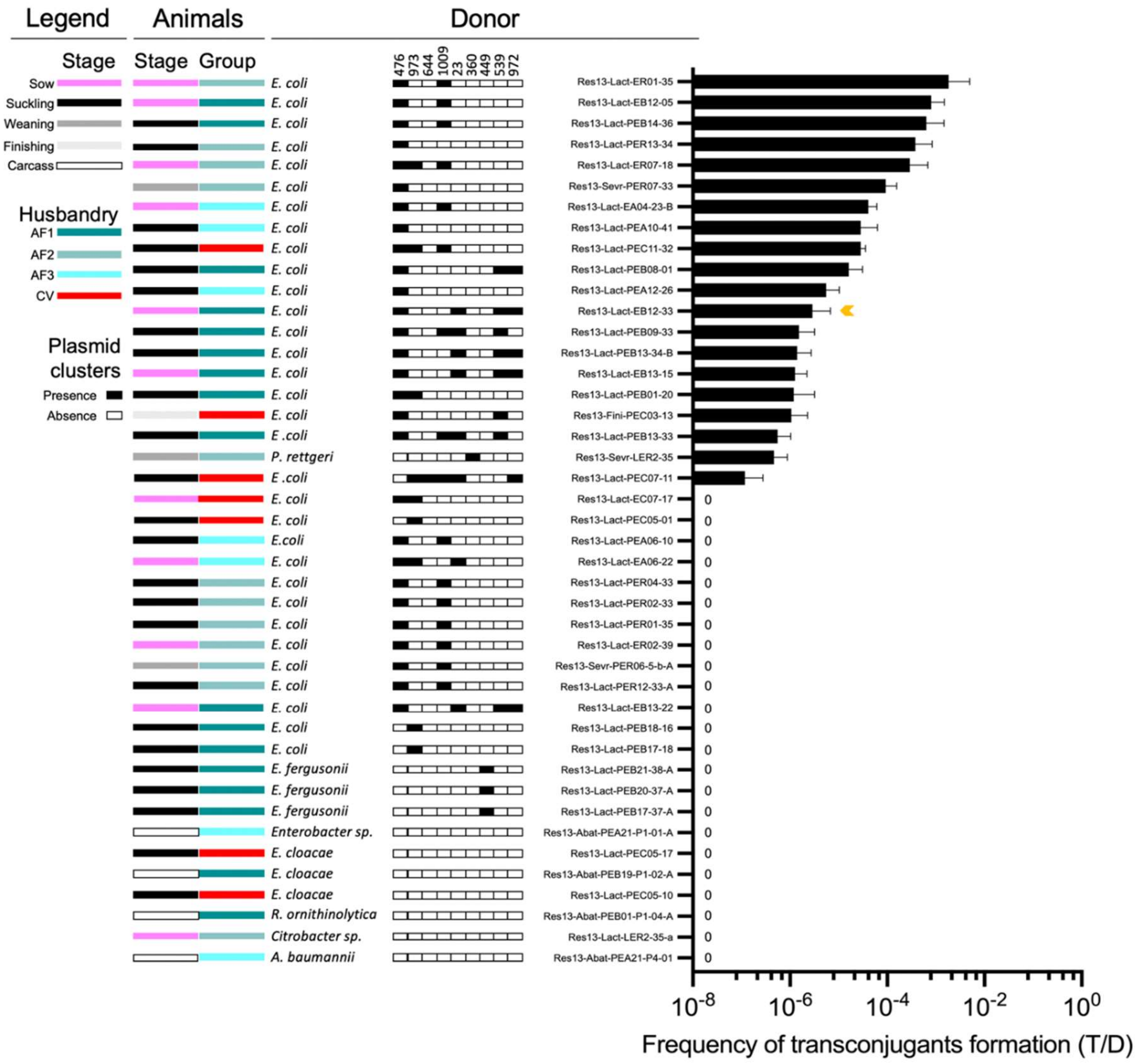

FIG S8 Propagation of cefotaxime resistance. Conjugation assays were carried out with cefotaxime-resistant isolates as donors and *E. coli* CV601 (KAN^R, RIF^R) as the recipient. The bars represent the means ± standard deviation of the frequencies of transconjugants formation calculated by dividing the number of transconjugants CFU (RIF^R, KAN^R, CTX^R) by the number of donors CFU (CTX^R). The animal stage and husbandry from which the donor isolates originated and the genera, plasmid cluster content (476, 973, 644, 1009, 23, 360, 449, 539, 972) and strain names are indicated. Only plasmids containing ARG were included. The orange chevrons indicate a mating from which a transconjugant was subjected to whole genome sequencing.

TABLE S5 Description of the animals sampled at each production stage.

Husbandry	Group	Sow[1]	Suckling	Weaning	Growing	Finishing	Carcass
Number of animals per group							
AF1	AF1-1	7	31	31	28	24	31
	AF1-2	7	29	-	-	-	-
	AF1-3	7	30	24	26	22	30
AF2	AF2-1	7	28	29	27	8	18
	AF2-2	7	30	34	27	11	22
AF3	AF3-1	7	34	32	-	-	-
	AF3-2	7	32	32	-	-	-
	AF3-3	7	32	27	35	30	38
	AF3-4	7	33	25	35	33	29
CV	CV-1	7	32	29	33	33	27
	CV-2	8	32	32	27	23	30
Days of lactation (sows) or age (piglets-pigs) at sampling							
AF1	AF1-1	14	14	35	84	182	189
	AF1-2	14	14	-	-	-	-
	AF1-3	14	14	35	84	182	189
AF2	AF2-1	14	14	35	84	182	189
	AF2-2	14	14	35	84	182	189
AF3	AF3-1	14	14	28	-	-	
	AF3-2	14	14	28	-	-	
	AF3-3	14	14	28	84	161	168
	AF3-4	14	14	28	84	161	168
CV	CV-1	14	14	28	84	161	168
	CV-2	14	14	28	84	161	168
Farm location[2]							
AF1	AF1-1	A	A	C	C	C	U
	AF1-2	B	B	-	-	-	-
	AF1-3	A	A	D	D	D	U
AF2	AF2-1	E	E	G	G	I	U
	AF2-2	F	F	H	H	I	U
AF3	AF3-1	J	J	L	-	-	-
	AF3-2	K	K	M	-	-	-
	AF3-3	K	K	N	O	P	U
	AF3-4	K	K	N	O	P	U
CV	CV-1	Q	Q	R	S	S	U
	CV-2	Q	Q	R	T	T	U

[1]Sows were at their 3rd parity except for sows of the AF1-2 group and one sow of the CV-1 group that were at their 2nd parity.
[2]A capital letter was attributed to each distinct location where the animals were housed. Location U was the abattoir.

TABLE S6 Distribution of isolates for each characterisation method.

Husbandry	Group	Sow	Suckling	Weaning	Growing	Finishing	Manure	Carcass	Feed
Number of isolates phenotyped by Sensititre™ automated microbroth dilution									
AF1	AF1-1	-	1	5	-	5	1	1	-
	AF1-2	9	7	-	-	-	-	-	-
	AF1-3	1	7	1	-	-	6	1	-
AF2	AF2-1	10	11	7	-	-	5	-	-
	AF2-2	5	7	-	-	5	7	-	-
AF3	AF3-1	11	1	-	-	-	1	-	-
	AF3-2	-	10	-	-	-	1	-	-
	AF3-3	13	10	17	15	11	-	7	-
	AF3-4	10	12	12	10	13	-	10	-
CV	CV-1	8	5	-	7	21	-	1	2
	CV-2	6	7	6	8	12	-	7	3
Number of whole genomes sequenced with Illumina Miseq									
AF1	AF1-1	4	6	8	5	9	1	1	-
	AF1-2	10	7	-	-	-	-	-	-
	AF1-3	1	7	4	2	3	6	1	-
AF2	AF2-1	8	11	8	4	2	5	-	-
	AF2-2	9	9	2	3	7	6	-	-
AF3	AF3-1	10	6	-	-	-	-	-	-
	AF3-2	4	10	1	-	-	1	-	-
	AF3-3	4	3	8	4	4	-	6	-
	AF3-4	4	4	3	3	8	-	10	-
CV	CV-1	10	7	-	3	10	-	4	-
	CV-2	4	2	7	3	2	-	9	-
Number of whole genomes sequenced with Oxford Nanopore MinION									
AF1	AF1-1	-	1	-	-	-	-	1	-
	AF1-2	4	5	-	-	-	-	-	-
	AF1-3	-	5	-	-	-	-	1	-
AF2	AF2-1	3	3	2	-	-	-	-	-
	AF2-2	1	5	1	-	-	-	-	-
AF3	AF3-1	1	1	-	-	-	-	-	-
	AF3-2	-	3	-	-	-	-	-	-
	AF3-3	-	-	-	-	-	-	1	-
	AF3-4	-	-	-	-	-	-	-	-
CV	CV-1	-	2	-	-	-	-	-	-
	CV-2	-	-	-	-	-	-	-	-

TABLE S6 Distribution of isolates for each characterisation method (continued).

Husbandry	Group	Sow	Suckling	Weaning	Growing	Finishing	Manure	Swab	Feed
Number of isolates tested as donor by conjugation.									
AF1	AF1-1	-	1	-	-	-	-	1	-
	AF1-2	4	5	-	-	-	-	-	-
	AF1-3	-	5	-	-	-	-	1	-
AF2	AF2-1	3	3	3	-	-	1	-	-
	AF2-2	-	2	-	-	-	-	-	-
AF3	AF3-1	2	1	-	-	-	-	-	-
	AF3-2	-	2	-	-	-	-	-	-
	AF3-3	-	-	-	-	-	-	2	-
	AF3-4	-	-	-	-	-	-	-	-
CV	CV-1	1	5	-	-	1	-	-	-
	CV-2	-	-	-	-	-	-	-	-

TABLE S7. Isolates and sequencing technical data. See joined Excel spreadsheet named TableS7.

References

1. CLSI. 2019. Performance Standards for Antimicrobial Susceptibility Testing. 29th ed. CLSI supplement M100. Clinical and Laboratory Standards Institute, Wayne, PA.

2. Robertson J, Nash JHE. 2018. MOB-suite: software tools for clustering, reconstruction and typing of plasmids from draft assemblies. Microb Genom 4:e000206.

3. Alcock BP, Raphenya AR, Lau TTY, Tsang KK, Bouchard M, Edalatmand A, Huynh W, Nguyen AV, Cheng AA, Liu S, Min SY, Miroshnichenko A, Tran HK, Werfalli RE, Nasir JA, Oloni M, Speicher DJ, Florescu A, Singh B, Faltyn M, Hernandez-Koutoucheva A, Sharma AN, Bordeleau E, Pawlowski AC, Zubyk HL, Dooley D, Griffiths E, Maguire F, Winsor GL, Beiko RG, Brinkman FSL, Hsiao WWL, Domselaar GV, McArthur AG. 2020. CARD 2020: antibiotic resistome surveillance with the comprehensive antibiotic resistance database. Nucleic Acids Res 48:D517-D525.
